# Single cell transcriptomic analysis of the canine duodenum in chronic inflammatory enteropathy and health

**DOI:** 10.3389/fimmu.2024.1397590

**Published:** 2024-06-12

**Authors:** Alison C. Manchester, Dylan T. Ammons, Michael R. Lappin, Steven Dow

**Affiliations:** ^1^ Colorado State University, Department of Clinical Sciences, College of Veterinary Medicine and Biomedical Sciences, Fort Collins, CO, United States; ^2^ Colorado State University, Department of Microbiology, Immunology and Pathology, College of Veterinary Medicine and Biomedical Sciences, Fort Collins, CO, United States

**Keywords:** single-cell RNA seq, duodenum, canine (dog), transcriptomics, chronic enteropathy

## Abstract

Chronic inflammatory enteropathy (CIE) is a common condition in dogs causing recurrent or persistent gastrointestinal clinical signs. Pathogenesis is thought to involve intestinal mucosal inflammatory infiltrates, but histopathological evaluation of intestinal biopsies from dogs with CIE fails to guide treatment, inform prognosis, or correlate with clinical remission. We employed single-cell RNA sequencing to catalog and compare the diversity of cells present in duodenal mucosal endoscopic biopsies from 3 healthy dogs and 4 dogs with CIE. Through characterization of 35,668 cells, we identified 31 transcriptomically distinct cell populations, including T cells, epithelial cells, and myeloid cells. Both healthy and CIE samples contributed to each cell population. T cells were broadly subdivided into GZMA^high^ (putatively annotated as tissue resident) and IL7R^high^ (putatively annotated as non-resident) T cell categories, with evidence of a skewed proportion favoring an increase in the relative proportion of IL7R^high^ T cells in CIE dogs. Among the myeloid cells, neutrophils from CIE samples exhibited inflammatory (SOD2 and IL1A) gene expression signatures. Numerous differentially expressed genes were identified in epithelial cells, with gene set enrichment analysis suggesting enterocytes from CIE dogs may be undergoing stress responses and have altered metabolic properties. Overall, this work reveals the previously unappreciated cellular heterogeneity in canine duodenal mucosa and provides new insights into molecular mechanisms which may contribute to intestinal dysfunction in CIE. The cell type gene signatures developed through this study may also be used to better understand the subtleties of canine intestinal physiology in health and disease.

## Introduction

Chronic inflammatory enteropathy (CIE) in dogs is a syndrome characterized by clinical signs indicating gastrointestinal (GI) tract dysfunction lasting longer than 3 weeks. Diagnosis is achieved by ruling out organic causes for these signs including infectious, endocrine, metabolic, and neoplastic causes (1). It is the most common reason for chronic diarrhea, vomiting, and/or poor appetite in dogs (2, 3), and is managed with various strategies including diet, microbiota modulators, and immunosuppressants (4). As such, canine CIE shares features with human irritable bowel syndrome (IBS), celiac disease, and inflammatory bowel diseases (5–7). Dogs and humans share dietary aspects and living environments which may contribute to disease, and thus spontaneously occurring CIE is a relevant model for enteropathies in humans, which have been increasing in prevalence (8). Advancements in understanding the pathogenesis of canine CIE are needed to improve outcomes, develop new therapies for dogs, and to enable the use of this condition as a translational model.

Histopathologic evaluation of intestinal biopsy samples is a common clinical tool used in the diagnostic workup and has also been employed to investigate CIE pathogenesis (9). Biopsies are characterized based on the severity of mucosal inflammatory cell infiltrates and morphological lesions of the epithelium and lamina propria (10). Updated grading schemes have attempted to reduce interobserver variability and subjectivity (11), but histopathologic evaluation of intestinal biopsies remains a tool of limited utility given overlap in histopathology findings between health and CIE (12, 13) and absence of specific lesions to guide clinical management (14, 15). Immunohistochemistry (IHC) has improved the granularity of histopathologic evaluations, enabling quantification of T cell subsets, including Foxp3-expressing regulatory T cells (12, 16). Despite these advances, the utility of IHC is restricted by limited pre-existing knowledge of cell markers of interest, and inability to look at numerous markers simultaneously. More sophisticated approaches are necessary to identify and characterize the diverse cell types present in the canine small intestinal mucosa.

Single-cell RNA sequencing (scRNA-seq) is a high-resolution molecular technique that enables the characterization of transcriptomic activity on an individual cell basis (17, 18). This approach overcomes traditional barriers to disease investigation (e.g., species-specific reagents) and enables characterization of rare cell subtypes among heterogeneous samples. Recently, two canine scRNA-seq reference datasets of peripheral blood mononuclear cells (PBMCs) and circulating αβ T cells have been generated, revealing numerous transcriptomically unique cell populations (19, 20). Application of scRNA-seq to celiac (21) and Crohn’s disease (22) have provided new molecular insights, and substantially advanced understanding of these complex conditions.

Given the translational relevance of canine CIE, we applied scRNA-seq to investigate the heterogeneity in canine duodenal mucosa to characterize cellular and molecular aspects of the disease. Through use of unsupervised clustering, cells were grouped according to transcriptomic similarity, and identities were assigned based on gene signatures. We identified 31 transcriptomically distinct cell subtypes, including intestinal epithelial, T cell, myeloid, and B cell populations. Our analysis revealed a skew in the relative proportions of T cell subsets, along with transcriptomic differences within myeloid and intestinal epithelial cells. Overall, our analysis provides valuable canine specific cell type gene signatures and provides important new insights into the pathobiology of CIE, from which further study can be completed.

## Methods

### Study population

Dogs with CIE were selected during screening for a prospective clinical trial at Colorado State University Veterinary Teaching Hospital (CSU-VTH) involving feeding of novel diet sourcing protein from individual amino acids (Colorado State University IACUC protocol #1440) (23).

Dogs were considered for inclusion in the CIE group if they (1) exhibited clinical signs compatible with chronic enteropathy for >3 weeks, (2) were not experiencing adequate relief from their CIE signs according to their owner, and (3) had a canine chronic enteropathy activity index (CCECAI) score ≥ 3. Extra-intestinal reasons for clinical signs were ruled out with a physical exam, complete blood count, chemistry panel, and assessment of serum cobalamin (B12), folate, canine trypsin like immunoreactivity (cTLI) and canine pancreatic lipase immunoreactivity (cPLI) concentrations. Exclusion criteria included serum albumin < 2.5 g/dL, treatment with antibiotics within 1 month of study enrollment, or immunosuppressive drugs within 7 days of GI biopsy collection.

Healthy dogs were selected from a colony of research beagles and were considered eligible for inclusion based on absence of clinical signs associated with CIE. The healthy beagles were screened with a physical exam, chemistry panel, and assessment of serum B12, cTLI and cPLI concentrations. Fecal samples from dogs had been screened with fecal flotation to identify intestinal helminth shedding within 2 months of sample collection.

### Sample acquisition

Dogs were fasted for up to 24 hours prior to endoscopy and anesthetized using routing protocols. The upper GI tract was inspected visually, and samples were collected from the stomach and duodenum for histopathology. In addition, 12 to 15 biopsies were collected from the proximal duodenum with single use standard 2.8 mm single-use biopsy forceps (Micro-Tech Endoscopy, Nanjing, China) then placed into ice cold Roswell Park Memorial Institute (RPMI) 1640 medium with L-glutamine (Corning, Glendale, AZ) until processing.

### Biopsy processing for single-cell RNA sequencing

Biopsy samples were rinsed with ice cold phosphate buffered saline (PBS), then centrifuged at 400 rcf for 5 minutes. To dissociate epithelial cells, biopsies were digested in Hanks’ balanced salt solution (HBSS; Gibco, ThermoFisher Scientific, Waltham, MA, USA) with 2 mM ethylenediaminetetraacetic acid (EDTA; ThermoFisher Scientific) and 10% fetal bovine serum (FBS; Peak Serum, CO, USA) for 30 minutes at 37°C with intermittent vortexing. Following the EDTA digestion, the supernatant was removed, passed through a 70 μM cell strainer (Greiner Bio-One, Monroe, NC), and stored in HBSS with 5% FBS and 10 mM 4-(2-Hydroxyethyl)-1-piperazineëthanesulfonic acid (HEPES) on ice. The remaining tissue was resuspended in HBSS with collagenase type II (Gibco, Grand Island, NY) at 250 U/mL and incubated for 30 minutes at 37°C with intermittent vortexing. The two cell fractions were pooled together then passed through a 40 μM cell strainer (Greiner Bio-One, Monroe, NC). The pooled cells were resuspended in 4 mls HBSS and layered on top of 3 mls of Ficoll-Paque PLUS (Cytiva, Uppsala, Sweden). Cells were isolated using density gradient centrifugation to enrich live cells for 30 minutes at 400 rcf with maximum acceleration and no brake. The cellular interface was aspirated and then washed with PBS. If cell yield was low, all cells were pooled to obtain adequate cell numbers. To remove contaminating red blood cells, cells were incubated in ammonium-chloride-potassium lysis buffer at room temperature for 3 to 5 minutes. A final 15-minute centrifugation at 100 rcf at 8°C was performed to remove small cellular debris and platelets. Lastly, cells were resuspended in 0.04% molecular grade bovine serum albumin (Sigma-Aldrich; St. Louis, MO) in PBS and transported to a Chromium iX instrument (10x Genomics; Pleasanton, CA) for cell capture. All samples were captured within 30 minutes of dissociation.

### Library preparation and sequencing

Single cells were isolated and tagged with molecular barcodes using a Chromium iX instrument with a target of 5,000 cells per sample (10x Genomics). Cellular cDNA was pooled and prepared for Illumina sequencing using Chromium Next GEM Single Cell 3′ Kit v3.1 and dual index library construction kits (10x Genomics). Library quality was analyzed using a LabChip (PerkinElmer; Waltham, MA) and sequenced on an Illumina NovaSeq 6000 sequencer (Novogene Corporation; Sacramento, CA) with a target of 50,000 150 bp paired-end reads per cell. Raw data were demultiplexed by the sequencing facility then transferred for downstream analysis.

### Read mapping and quantification

A Cell Ranger analysis pipeline (version 6.1.2, 10x Genomics) was utilized to process raw FASTQ sequencing data, align reads to the canine genome, and generate a count matrix. The default settings were used when running “cellranger count” and aligned to a CanFam3.1 (Ensembl release 104) reference prepared as previously described (24). To obtain TRDC expression the three healthy samples were aligned to an alternate canine genome (ROS_Cfam_1.0; Ensembl release 111) using the same alignment protocol that was used to align the data to CanFam3.1.

### Data filtering, integration, dimension reduction, and unsupervised clustering

The count matrix for each sample was imported into R (version 4.1.1) using the Read10X() function then converted to a Seurat object (version 4.3.0) using the CreateSeuratObject() function (25). To estimate the number of dead/poor quality cells, the percentage of mitochondrial reads per cell was calculated using PercentageFeatureSet() to count all reads mapped to features with the prefix “MT-”. Consistent with previous reports of scRNA-seq in human duodenal cells, we observed epithelial populations to have greater than 60% mitochondrial reads (26, 27). To account for the difference in mitochondrial feature percentages within immune and duodenal populations we completed two filtering steps to prepare the dataset for analysis. First, each object was filtered leniently to allow for retention of all cells except high end outliers using the following parameters: UMI counts per cell (100 < nCount_RNA < 25000), unique feature counts per cell (200 < nFeature_RNA < 3000), and percentage of mitochondrial reads per cell (percent.mt < 80). Then, DoubletFinder (version 2.0.3) was used to identify and remove putative cell doublets (28). Following doublet removal, a second filtering step was applied to non-epithelial cells in which cells with greater than 12.5% of reads mapping to mitochondrial features were excluded from downstream analysis. After completing QC filtering on each sample, all samples were integrated into one object using a SCTransform() normalization and canonical correlation analysis (CCA) integration workflow with 2500 features selected as integration anchors (25). During this step, we used the percent mitochondrial reads as a latent variable in a linear regression framework to minimize the impact of mitochondrial reads on dimension reduction and integration. Following data integration, one additional low-quality cluster (defined by low UMI counts) was identified and removed. Ideal clustering parameters for the complete dataset (res = 1.3 dims = 40, n.neighbors = 50, min.dist = 0.3) and the parameters for each cell type subset were determined using the R package clustree (version 0.4.4) (29). Dimension reduction and visualization was completed, and the data were projected using 2-dimensional, non-linear uniform manifold approximation and projection (UMAP) plots.

In addition to the standard workflow described above, we also employed a single-cell Variational Inference (scVI) integration approach as a supplementary method for analysis of the T cell subset (30). This approach uses variational inference and stochastic optimization of deep neural networks to transform the data into a low-dimensional space. Relative to the Seurat’s CCA approach (used as our primary method), scVI integration can better correct batch effects and is more accurate in partitioning cells into neighborhoods. A separate computing environment was used to complete scVI (version 1.1.1) integration in which the integrateData() function from Seurat (version 5.1.0) with the “scVIIntegration” method was employed. Following integration, the data were projected onto a UMAP embedding as described above.

### Cell classification

Following unsupervised clustering, the FindAllMarkers() function in the Seurat package was used to identify the enriched features in each cluster relative to all others (test.use = “wilcox”, only.pos = TRUE). The gene lists were manually evaluated to identify enrichment of canonical markers as defined in canine and human cell types. Key features and references supporting the use of specific markers to define a cell type are presented in **Supplementary Table 1**. To supplement manual annotation, we also used SingleR (31) and Seurat’s reference mapping protocol to transfer cell type annotations from a canine leukocyte atlas and the human gut cell atlas (19, 32). Following manual and algorithmic cell type identification, the cell type divisions derived from unsupervised clustering were then collapsed into biologically relevant cellular subtypes. Both the collapsed clustering (primary figures) and results of unsupervised clustering (supplemental) are presented. This process was completed on the full integrated dataset, as well as each of the subsets analyzed through subset analysis, and the approach used clustree output at multiple clustering resolutions to inform the identification of parent clusters.

### Feature visualization

Gene expression was visualized using (1) colorization of expression (log transformed data) values on UMAP plots, (2) dot plots of expression (scaled, log transformed data) by cluster, or (3) violin plots of expression (log transformed data) by cluster. Expression of features between conditions (CIE versus healthy) were visualized by splitting the UMAP (or dot plot) by condition then plotting the expression values. When completing visualization with a split UMAP plot, the conditions were down sampled to obtain equal cell numbers depicted in the UMAP plot for each condition.

### Cell abundance analysis

Cell type percentages were determined for each sample with the denominator being the total number of cells present in the subset for a given sample. The cell type percentages were then used to complete a two-sided Wilcoxon rank-sum test to evaluate statistical significance between samples from CIE and healthy tissues. The P value threshold for statistical significance was 0.1. Where applicable, the mean percentage ± standard deviation is provided for cells from healthy and CIE samples. In addition to statistical approaches, the data were visually inspected in the UMAP embedding through use of colorization by sample as well as plotting using stacked bar graphs. For stacked bar graphs, the depicted percentages were calculated using the percentage of cells from a given sample out of all cells within a given cluster. To avoid bias from differences in cell numbers, all biological replicates were down sampled to obtain equal representation prior to the calculation of percentages.

As a supplemental approach for the analysis of T cells, we used miloR (version 1.10.0) on scVI integrated data to evaluate differential abundance at the neighborhood level (33). This analysis has increased sensitivity as it evaluates abundance skews in hundreds of neighborhoods instead of a few annotated cell type clusters. Briefly, we generated a neighborhood by sample count matrix containing the numerical counts of cells then used the GLM framework in edgeR (version 4.0.14) to evaluate differential abundance (34). miloR was used to calculate a spatially corrected adjusted P value and statistical significance was evaluated at a SpatialFDR < 0.2.

### Differential gene expression analysis

Gene expression changes between cells were evaluated using pseudobulk conversion followed by a DEseq2 (version 1.34.0) workflow (35). This approach consisted of removing features that had less than 10 cells with non-zero expression values then collapsing raw counts data for each sample into one column. Only clusters with at least 15 cells were included in the pseudocount matrix. Statistical testing was then conducted differently depending on the application within the study. For analysis comparing gene expression between two cell type clusters (i.e. cluster 1 versus cluster 2 within the T cell subset) the P values were determined by testing the null hypothesis that | log2(Fold change) | < 1. Features were then considered to be significantly differentially expressed if the adjusted (FDR) P value was less than 0.01. For analysis comparing cells from CIE to healthy tissues each P value was determined by testing the null hypothesis that | log2(Fold change) | = 0. Features were then considered to be significantly differentially expressed though *post-hoc* filtering, such that significant genes were defined as adjusted (FDR) P value < 0.1 and | log2(Fold change) | > 1.

### Gene set enrichment analysis

Statistically significant differentially expressed features (between conditions or clusters) were used as input to complete gene set enrichment analysis using the enricher() function from the clusterProfiler R package (version 4.2.2) (36). The “C5”, “C2”, and/or “Reactome” databases from the msigdbr package (version 7.5.1) were used to complete enrichment analysis (37). Significance was evaluated based on terms that achieved an FDR corrected P value < 0.05. Enriched terms were plotted using the signed log10(adjusted P value) to depict significance.

### Flow cytometric analysis

Remnant cells from the samples used for scRNA-seq were analyzed using flow cytometry to interrogate immune cell protein expression. Approximately 500,000 cells per sample were used to complete immunostaining. Each sample of dissociated duodenal cells was stained with a panel of 4 directly conjugated antibodies (CD45, CD11b, CD4, CD5; **Supplementary Table 2**). Cells were washed with FACs buffer (5% FBS with 0.1% sodium azide in PBS), blocked with 5 μL normal dog serum (Jackson ImmunoResearch Labs, West Grove, PA), then incubated in the primary antibodies diluted in FACS buffer for 25 minutes at 4°C. Cells were washed with FACS before 5 μL 7-AAD (Invitrogen, Waltham, MA, USA) was added, and samples were run on a Beckman Coulter Gallios 3-laser flow cytometer. Flow data were analyzed with FlowJo software version 10.8.1. The gating strategy is displayed in **Supplementary Figure 1**.

### Data and software availability

Raw sequencing data and cell by gene count matrices are available on the NCBI Gene Expression Omnibus database (GSE254005). The annotated dataset is available for browsing on the UCSC Cell Browser (https://cells.ucsc.edu/?ds=canine-duodenum-cie) (38). Processed data (Seurat RDS objects), analysis code, and software versions are available on Zenodo and at https://github.com/dyammons/canine_duodenal_atlas (24). Any additional data requests can be made by contacting the corresponding author.

## Results

### Patient populations

The healthy dog cohort consisted of 3 adult colony intact male beagles. Physical exam and diagnostic test results (chemistry panel, fecal parasite screening) were unremarkable, and all dogs had normal serum cobalamin concentrations (**Table 1**). The CIE dogs included dogs of distinct breeds and inadvertently were all neutered males. Clinically, all presented with chronic diarrhea (**Table 1**) and canine chronic enteropathy clinical activity indices (CCECAI) compatible with mild to severe disease (15). Physical exam findings in the CIE dogs included low body condition score in 2 dogs with normal body condition scores in the other 2. Two dogs were hypocobalaminemic (serum vitamin B12 <250 ng/L).

**Table 1:** Study dog characteristics. CCECAI, canine chronic enteropathy clinical activity index.

	ID	age (y)	sex	breed	weight (kg)	BCS	CCECAI	albumin (g/dL)	B12 (ng/L)	medications
Healthy	H_1	6	M	Beagle	14.4	5	0	3.5	525	none
	H_2	5	M	Beagle	10.4	5	0	4.3	492	none
	H_3	6	M	Beagle	13.4	5	0	3.7	763	none
CIE	CIE_1	5.5	MN	Great Pyrenees	39	3	7	3.3	227	none
	CIE_2	6.5	MN	Standard poodle	31.3	5	3	3.5	352	phenobarbital 3.1 mg/kg PO q12h, alprazolam 3.2 mg/kg PO q24h, gabapentin 9.6 mg/kg PO q12h, Visbiome Vet
	CIE_3	2	MN	Great Dane	25.7	1	13	3.0	145	mirtazapine 1.1 mg/kg PO q12h, Visbiome Vet, Fortiflora
	CIE_4	2.5	MN	Labrador retriever	29.1	5	9	3.1	>1000	cyanocobalamin 1000 μg PO q24h

BCS, body condition score (based on scale of 1 to 9).

**Figure 1:**
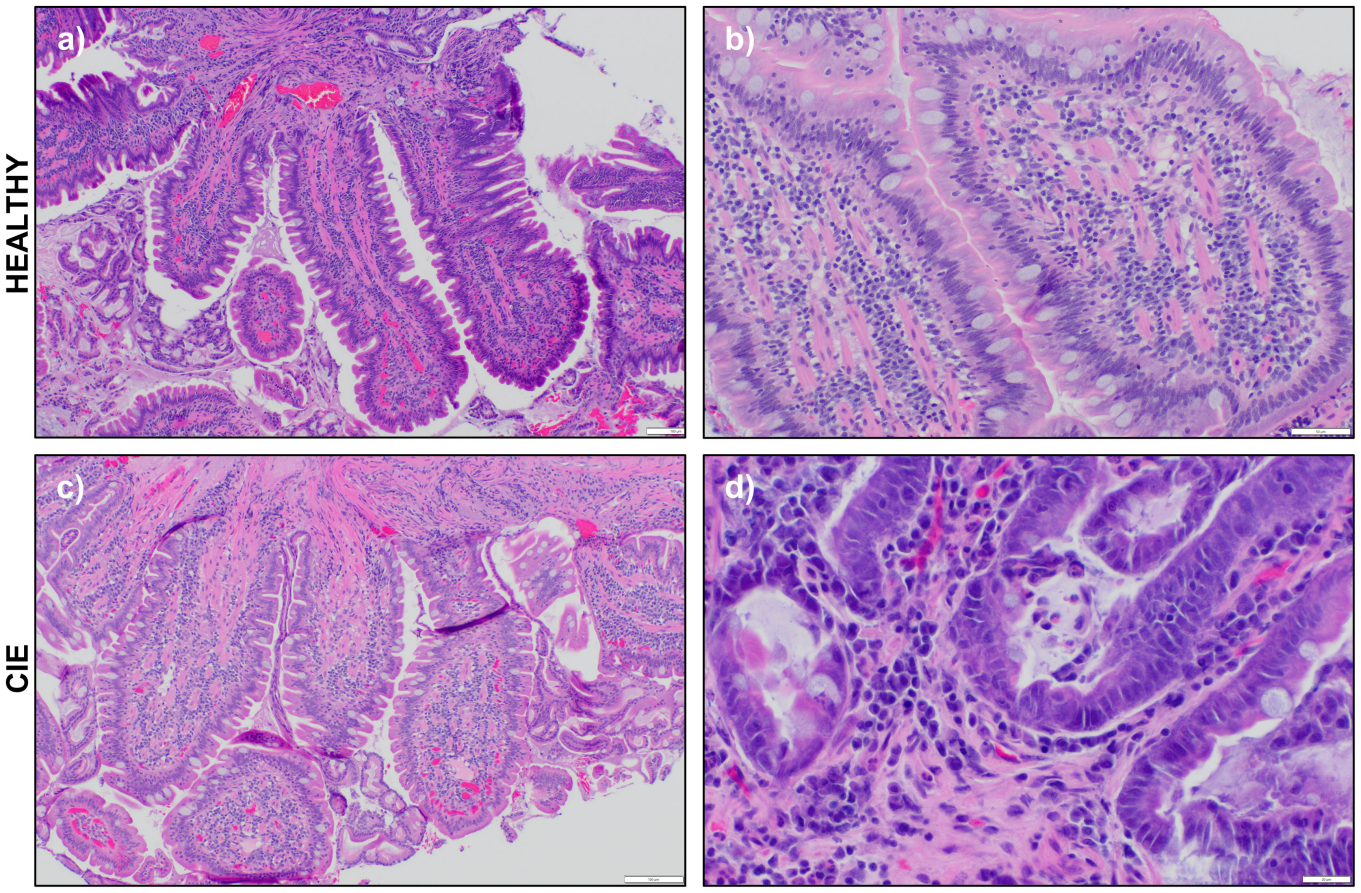
Representative photomicrographs of hematoxylin and eosin-stained duodenal biopsies. Photomicrographs of endoscopically obtained duodenal biopsies viewed at 10X, 40X, or 100X magnification from **(A, B)** a healthy dog (H_1) and two CIE dogs **(C)** CIE_4 and **(D)** CIE_3. Scale bar indicates 100 **(A, C)**, 50 **(B)** or **(D)** 20 μm.

Duodenal biopsies from all study dogs were evaluated with routine histopathology by a single board-certified veterinary pathologist, and results are provided in **Supplementary Table 3**. Samples from healthy dogs revealed evidence of mild (n=1) to moderate (n=2) lymphoplasmacytic (LPL) infiltrates with no morphologic lesions detected (**Figures 1A, B**). One healthy dog had a mild neutrophilic infiltrate. These dogs were retained as healthy controls as the dogs lacked clinical signs of GI disease and LPL duodenal infiltrates are not specific for clinical disease (13, 39–41). Histopathologic conclusions in the CIE dogs included LPL enteritis with mild (n=3) to marked (n=1) neutrophilic infiltrates (**Figure 1C**). A marked eosinophilic component, along with mild crypt dilation and epithelial surface injury, was also appreciated in one dog (CIE_3; **Figure 1D**).

### Generation of a cellular atlas from canine duodenal mucosa

The first objective of this study was to generate an atlas of cells present in the adult canine duodenal mucosa. To accomplish this goal, we profiled the transcriptomes of 35,668 cells from duodenal biopsies obtained from 3 healthy and 4 CIE dogs. The average number of cells collected from each study dog was 5,095, and each sample was sequenced to an average depth of 43,956 reads per cell (**Supplementary Table 4**). Cell viability assessed by flow cytometric analysis indicated >95% live cells in each sample (**Supplementary Table 5**).

**Figure 2:**
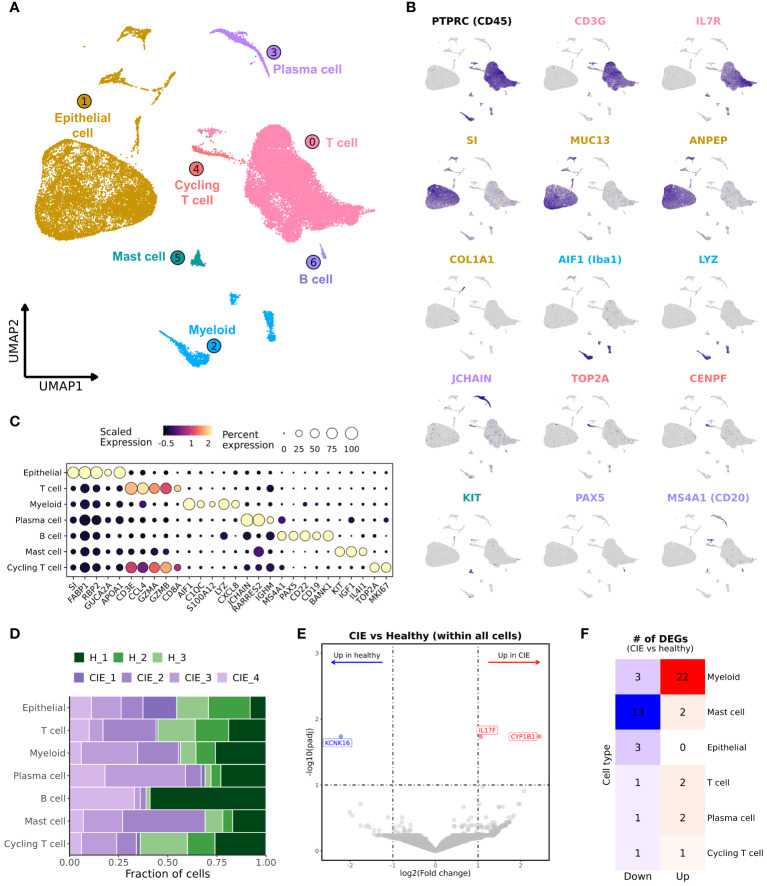
Unsupervised clustering of duodenal endoscopic biopsies from 3 healthy and 4 CIE affected dogs separates cells into 7 distinct populations. **(A)** UMAP representation of 35,668 cells from dissociated duodenal biopsies from 7 dogs. **(B)** Feature plots depicting expression of select genes associated with the identities of the major cell types. **(C)** Dot plot representing scaled, log transformed gene expression for each major cell population. Dot size represents the percentage of cells expressing the gene while dot color represents relative expression. **(D)** Stacked bar graph representing contributions to the 7 major cell populations by the 3 healthy dogs (greens) and 4 CIE dogs (purples). **(E)** Volcano plot depicting results of differential gene expression analysis, in which log2(Fold change) is depicted on the x-axis and the -log10(adjusted P value) is on y-axis. The significantly enriched (red) and downregulated (blue) genes in CIE relative to healthy are labeled. **(F)** Heatmap depicting the number of differentially expressed genes, CIE compared to healthy, within each major cell type. Features are included in counts if adjusted P value < 0.1 andlog2(Fold change)> 1. Features with a positive log2(Fold change) are in right column (“Up”), while features with a negative log2(Fold change) are in left column (“Down”).

Analysis of the integrated dataset revealed the presence of 7 major cell types which included epithelial cells, T cells, myeloid cells, mast cells, B cells, plasma cells, and a population of cycling T cells (**Figure 2A**; **Supplementary Data 1**). Through subcluster analysis of the major cell populations, we were able to further divide the dataset into 31 transcriptomically distinct populations (**Table 2**; **Supplementary Figure 2A**; **Supplementary Data 2**). Annotation of the major cell types was completed using canonical markers, with reference mapping to human scRNA-seq duodenal data and a canine scRNA-seq leukocyte dataset (**Figures 2B, C**; **Supplementary Table 1**; **Supplementary Figures 2B–D**) (19, 32). Briefly, epithelial cells were characterized by expression of sucrase isomaltase (SI), fatty acid binding protein 1 (FABP1), retinol binding protein 2 (RBP2), and apolipoprotein A1 (APOA1) (27, 32, 42). T cells were identified based on expression of CD3E, CCL4, PTPRC (CD45) and IL7R (43, 44). Myeloid cells included cells exhibiting overexpression of allograft inflammatory factor 1 (AIF1), lysozyme (LYZ) and complement protein genes (C1QA, C1QB, C1QC) (32, 45). Plasma cells were enriched in expression of JCHAIN and retinoic acid receptor responder 2 (RARRES2) expression (19, 42), while B cells were defined by MS4A1 (CD20), CD19, and PAX5 expression (19, 32, 45). Mast cells exhibited relative overexpression of KIT, IGF1 and CD52 (21, 45). Lastly, the population of cycling T cells was defined by expression of T cell markers with additional expression of TUBA1B, TOP2A and STMN1 (42, 45). To assess the accuracy of major cell type annotation, we evaluated the correlation of relative cell type proportions as determined by scRNA-seq with the proportions determined using flow cytometry (**Supplementary Figure 2E**). The analysis revealed a strong correlation (R^2 ^= 0.95) with a slope of 0.97, indicating concordance of cell type classifications between the two approaches.

**Table 2:** Top upregulated genes comprising transcriptional signatures of canine duodenal cells.

Cell type	Major markers
T cells	
Effector CD8 (CD8_eff)	GZMA, GZMB, CCL5, CD7, CD2, ITGB7, CD96, CD3E
Naïve (Tnaive)	VIM, IL7R, S100A8, EEF1A1, CXCR4, LTB, TMSB10
Tissue resident memory (CD8_TRM)	CCL4, RGS1, IFNG, NR4A2, BCL2A1, FASLG
γδ T subset 1 (gdT_1)	GZMA, TRAT1, GZMB, TNFRSF6B, PTPN22, CCL5
Natural killer T (NK_T)	CRTAM, CTSW, CD160, TCF7, TESC, NME1, REL
Memory CD8 (CD8_mem)	GZMK, FOS, SH2D1A, CCL4, CXCR4, KLF4, DLA-DRA
γδ T subset 2 (gdT_2)	ENSCAFG00000030206, CCL5, SPNS3, GZMA, CAPG, IL2RB, TRAT1, CD7
Regulatory T (Treg)	TNFRSF4, TNFRSF18, S100A5, CTLA4, ICOS, NFKBIA
Natural killer (NK)	KRLB1, NFKBID, NCR3, F2RL3, IL12RB2, CTSW
Interferon-enriched T (T_IFN)	ISG15, MX2, IFGGB2, OAS1, IFI44, SAMD9L, IFI44L
type 2 innate lymphoid (ILC2)	MMP9, IL17RB, GATA3, PLAC8A, CLINT1, MPP4, ARL4C
CD20^+^ T (MS4A1_T_cell)	MS4A1, CD52, TESC, FCER1G, STAP1, GZMB, IL2RB
Epithelial cells	
Enterocyte 1	ND4L, UGT1A6, SI, MYO1A, FAM13A, FAM15A, SLC22A4, SLC43A2
Enterocyte 2	APOC3, APOA4, APOA1, APOB, TM4SF20, CLCA4, SLC40A1, LGALS3
Enterocyte 3	FABP1, ATP5MC1, MGST3, COX4I1, RBP2, GSTP1, SLC25A5, SLC25A6
BEST4+ enterocyte	GUCA2A, GUCA2B, CFTR, SYNC, STOM, CA7, BEST4
Goblet cell	ZG16, SPINK4, CLCA1, BCAS1, LYPD8, PNLIP, SYTL2
Tuft cell	ANXA4, CA2, TRPM5, IRAG2, RYR1, FYB1, SYNJ1, ATP2A3
Interferon-enriched enterocyte (IFN_enterocyte)	ISG15, RSAD2, IFIT1, DDX60, APOC3, RNF213, OAS1, SLC40A1
Stromal cell	TPM2, VIM, ADAMDEC1, IGFBP7, COL1A2, MYL9
Enteroendocrine cell	CHGB, ADGRG4, SCG2, AFP, UNC13B, PCSK1N, TPH1
Myeloid cells	
Neutrophil	S100A12, S100A8, SERPINA1, SOD2, NFKBIA, SELL, ISG20
Eosinophil	MMP1, MS4A2, CAT, PGF, CHI3L1, ADAMDEC1, FABP3
Monocyte	MT1E, MT2A, HMOX1, C1QC, C1QA, GSTP1, CTSS
Macrophage	CCL3, MAFB, C1QA, C1QC, STAB1, CTSS, CCL7, C1QB
cDC1	TMSB10, BATF3, ECRG4, CLEC1B, NAPSA, DNASE1L3
IL22RA2 DC	IL22RA2, TMBS10, CD52, ECRG4, MAL, S100A5, FBP1, CA2, LSP1
Miscellaneous	
**Plasma cells**	JCHAIN, IGHM, TXNDC5, RARRES2, PRDX4, DERL3
**Cycling T cells**	TUBA1B, TOP2A, STMN1, CENPF, HMGB2
**Mast cells**	KIT, IGF1, CD52, CXCR4, SVIL, F2RL3
**B cells**	MS4A1, TNFRSF13C, DLA-DRA, FCRLA, SAMSN1, CD22, PAX5

Evaluation of cell type abundances revealed marked variability within both the healthy and CIE dogs (**Figure 2D**; **Supplementary Figures 3A, B**, **4**). Statistical evaluation of major cell type proportions revealed no significant differences between conditions (**Supplementary Figure 3B**). Given this finding, we next completed differential gene expression (DGE) analysis within the full dataset between CIE and healthy dogs (**Figure 2E**; **Supplementary Data 3**). This analysis revealed few differentially expressed genes (DEGs), of which CYP1B1 and IL17F were enriched, and KCNK16 was downregulated in dogs with CIE. Evaluation of expression within each major cell type indicated differential expression of KCNK16 and CY1B1 was related to epithelial cells, whereas IL17F was specific to mast cells (**Supplementary Figure 3C**). To further explore the origin of CIE associated differences, we completed DGE analysis within each major cell subset. This analysis identified the greatest number of DEGs in myeloid, mast cells, and epithelial cells, while the fewest number of DEGs were found within plasma cell, T cell, and cycling T cell populations (**Figure 2F**; **Supplementary Data 3**). From here, we investigated each major cell type individually to complete a more refined evaluation of the dataset.

### Neutrophilic gene signature distinguishes healthy from CIE duodenal mucosa

**Figure 3:**
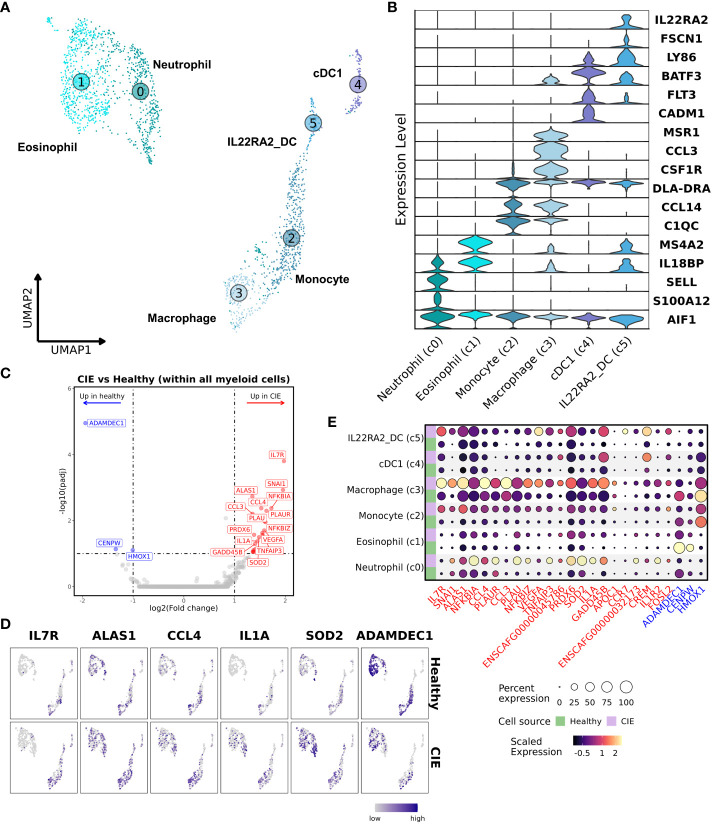
Subset analysis of myeloid cells highlights the presence of pro-inflammatory neutrophils in duodenal mucosa of CIE dogs. **(A)** UMAP representation of unsupervised clustering of 1,945 myeloid cells. Myeloid cell subtypes are labelled based on assigned identity, with increasing numbers corresponding to smaller relative contribution to the myeloid pool. Neutrophil (c0); eosinophils (c1); monocytes (c2); macrophages (c3); cDC1 (c4; conventional dendritic cell type 1); IL22RA2_DC (c5; IL22RA2 expressing dendritic cells). **(B)** Violin plots of key features defining each myeloid cell subtype. **(C)** Volcano plot depicting results of differential gene expression analysis within all myeloid cells, in which log2(Fold change) is depicted on the x-axis and the -log10(adjusted P value) is on y-axis. The top 20 (weighted by adjusted P value) significantly enriched (red) and downregulated (blue) genes in CIE relative to healthy are labeled. **(D)** Split UMAP plots of select differentially expressed genes in duodenal myeloid cells from healthy (top pane) or CIE (bottom pane) dogs. **(E)** Split dot plot representing scaled, log transformed gene expression of top upregulated (red text) and downregulated (blue text) genes within each myeloid cell subpopulation. Dot size represents the percentage of cells expressing the gene while dot color represents relative expression. Green rows display contributions from healthy dogs while purple rows display contributions from CIE dogs.

Analysis of AIF1 (Iba1) expressing cells revealed the presence of 6 myeloid cell subtypes: neutrophils, eosinophils, monocytes, macrophages, and 2 dendritic cell (DC) populations (**Figures 3A, B**; **Table 2**; **Supplementary Figure 4A**; **Supplementary Data 4**). Granulocytes (c0 and c1) were classified based on a lack of DLA-DRA (MHCII) expression with expression of MS4A2 or S100A12 in the eosinophil and neutrophil clusters, respectively (46). Monocyte, macrophage, and the two DC clusters were defined by DLA-DRA expression, with macrophages expressing MSR1 (CD204), monocytes lacking MSR1 expression, and DCs expressing FLT3 (47). Macrophages also exhibited preferential expression of CD163, CD64 (FCGR1A), phagocytosis-associated genes (ANXA1, MERTK, NR1H2, NR1H3), and features compatible with intestinal resident macrophages (48, 49). The FLT3 positive cells were divided into two populations, a conventional dendritic cell type 1 (cDC1) population (19) and a distinct DC population defined by expression of IL22RA2 and FSCN1. Statistical evaluation of myeloid cell subtype proportions revealed no statistically significant differences between the CIE and healthy dog samples (**Supplementary Figures 4B-D**).

Subsequently, we completed DGE analysis to evaluate transcriptomic differences across all myeloid cells from healthy and CIE dogs. This analysis demonstrated a marked increase in the expression of genes related to inflammatory mediators (IL1A, SOD2, TNFAIP3) in CIE affected dogs (**Figure 3C**). Gene set enrichment analysis (GSEA) of genes upregulated in CIE revealed an enhancement of pathways associated with interleukin signaling and reactive oxygen species (**Supplementary Figure 4E**), suggesting an altered inflammatory state in CIE. Further investigation on an individual sample basis revealed CIE_3 contributed disproportionally to expression of the identified DEGs relative to other CIE dogs (**Supplementary Figure 5**). This finding was consistent with histopathologic evaluation, whereby a marked neutrophilic and eosinophilic infiltrate was identified in CIE_3, but not in the other CIE dogs (**Figure 1D**; **Supplementary Table 3**). Visualization of top DEGs in the UMAP embedding and through use of dot plots confirmed that most of the DEGs stemmed from increased expression within the neutrophil cluster, while also highlighting the role of the macrophage cluster (**Figures 3D, E**). This distribution suggests that duodenal mucosal neutrophils and macrophages exhibit pro-inflammatory programs in CIE dogs relative to healthy dogs. In summary, myeloid cells represented a small fraction (5.5%) of total duodenal mucosal cells in this dataset, but transcriptional differences suggest that pro-inflammatory neutrophils may be enriched in the duodenum of some dogs with CIE.

### Subset analysis of duodenal T cells reveals 10 cell subpopulations with evidence of a CIE associated shift in T cell subtype proportions

**Figure 4:**
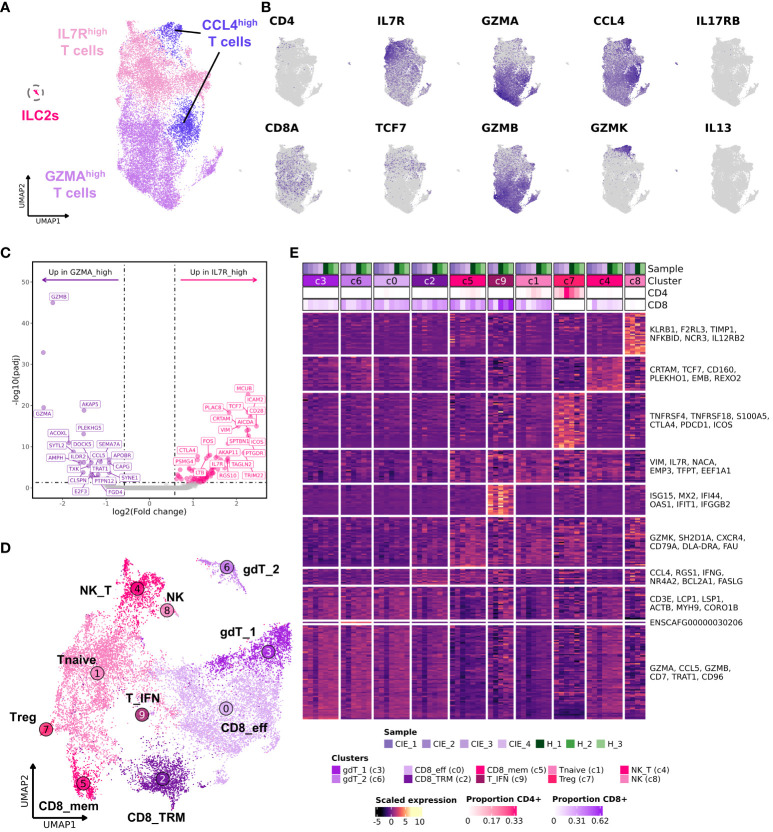
Subcluster analysis of duodenal T cells reveals 10 distinct cell subpopulations. **(A)** UMAP representation of low-resolution unsupervised clustering of 18,824 T cells. Cells are divided into 4 broad categories: IL7R^high^, GZMA^high^, CCL4^high^ and innate lymphoid cells (ILC2s). **(B)** Feature plots displaying expression of select genes. **(C)** Volcano plot displaying differentially expressed genes contrasting IL7R^high^ T cells to GZMA^high^ T cells based on DESeq2 analysis. The top 20 (weighted by adjusted P values) upregulated (pink) and downregulated (purple) genes are labeled. **(D)** UMAP representation of high-resolution cell type annotations for duodenal T cells excluding ILC2s. **(E)** Heat map depicting scaled gene expression of the 10 T cell subtypes with individual dogs on columns and genes on rows. The top 6 genes (weighted by adjusted P value) identified using a Wilcoxon rank-sum test are labeled. The proportion of CD4 or CD8 positive cells within each cluster is depicted in the top annotation of the heatmap. IL7R^high^ T cells: [Tnaïve (c1), naïve T cells; NK_T, NK T cells (c4); Treg, regulatory T cells (c7); NK, natural killer T cell (c8); T_IFN, interferon signature T cells (c9)]. GZMA^high^ T cells: [CD8_eff, CD8 resident effector T cells (c0); gdT_1, gamma delta T cell 1 (c3); gdT_6, gamma delta T cell 2 (c6)]. CCL4^high^: [CD8_mem, CD8 memory T cells (c5); CD8_TRM, CD8^+^ tissue resident memory T cells (c2)].

Through low-resolution unsupervised clustering of T cells (n = 18,824) we identified 4 transcriptomically distinct populations (**Figure 4A**; **Supplementary Data 5**). Clustering of T cells was not overtly driven by CD4/CD8 expression, as evidenced by varied expression of those features within each cluster (**Figure 4B**). Thus, we sought alternative means of distinguishing and annotating the T cell populations (**Figure 4B**; **Supplementary Figure 6A**). The cluster annotated as IL7R^high^ T cells was enriched in IL7R, TCF7, CXCR4, SELL, and CCR7 expression, which is compatible with recently circulating T cells that have not yet established residency in the duodenal mucosa (50, 51). The cluster annotated as GZMA^high^ T cells exhibited overexpression of effector molecules (GZMA, GZMB) and integrins (ITGB7, ITGAE) which is suggestive of an intestinal mucosal tissue resident T cell phenotype (52–54). To further compare the two broad subdivisions of T cells, we completed DGE analysis to identify the features that defined each population (**Figure 4C**; **Supplementary Data 6**). Subsequent GSEA revealed IL7R^high^ T cells to be enriched in terms associated with immune cell differentiation and adhesion (**Supplementary Figure 6B**), further supporting the classification as a non-resident T cell subtype. Unsupervised clustering also revealed the presence of a population of cells expressing high levels of CCL4 split between the UMAP regions of IL7R^high^ T cells and GZMA^high^ T cells (**Figure 4A**). This clustering pattern suggested that both groups of T cells had a subpopulation of memory cells expressing this T cell recruitment molecule, with the population in the IL7R^high^ region also being defined by overexpression of an additional memory associated feature, GZMK (55). The final major T cell population identified in our initial analysis was innate lymphoid type 2 (ILC2) cells. These cells clustered separately and were defined by IL13, IL17RB, and GATA3 expression (56, 57). Reference mapping to the Human Gut Atlas further supported the ILC2 annotation (**Supplementary Figures 6C-E**) (32).

To further investigate the reliability of these findings, we re-analyzed the T cell dataset using a single cell Variational Inference (scVI) integration approach (**Supplementary Figure 7**) (30). This alternative approach, which uses stochastic optimization and deep neural networks, was chosen because it is reported to be a robust method that can efficiently capture biologically relevant cell subtypes. We found substantial overlap between the clustering results of Seurat’s CCA approach and scVI, but scVI enabled clearer separation between cells based on CD8 and CD4 expression (**Supplementary Figures 7A-E**). Furthermore, the analysis captured a population of MS4A1 (CD20) and TRDC expressing T cells which clustered distinctly in the full dataset UMAP embedding (**Supplementary Figures 2A**, **7A, E**), but were unidentifiable in the CCA integrated T cell UMAP embedding (**Figures 4A, D**). Although CD8^+^CD20^+^ T cells have been reported in the context of human colorectal cancer (58), there is a possibility that this cluster represents T-B cell doublets, so we refrain from drawing any conclusions regarding the population.

With major T cell populations identified, we next filtered out ILC2s and repeated subcluster analysis at a higher resolution to better study the T cell subpopulations in the dataset (**Figures 4D, E**; **Supplementary Figures 8A, B**; **Supplementary Data 7**). This analysis revealed that the IL7R^high^ T cells could be further subdivided into 6 subpopulations, including cells consistent with regulatory T cells (Tregs), natural killer (NK) cells, NK-T cells (NK_T), memory CD8 T cells (CD8_mem), T cells enriched in interferon gene signatures (T_IFN), and naïve T cells (T_naive). Subcluster analysis within the GZMA^high^ T cells revealed 4 subpopulations which included two CD8 T cell populations annotated as CD8 tissue resident memory (CD8_TRM) and CD8 resident effectors (CD8_eff), and two groups of γδ (gamma-delta) T cells which were annotated based on the expression of TRDC (gdT_1) or ENSCAFG00000030206 (TRGC2 ortholog) (gdT_2) (**Supplementary Figure 8C**). Notably, the UMAP embedding of the scVI integrated data condensed the two γδ T cell subsets into one cluster intermixed with CD8 T cells (**Supplementaryry Figure 7E**). This finding emphasized the relatedness of γδ T cells and CD8 T cells in dogs and provides evidence that two γδ T cell subsets identified using Seurat’s CCA are likely more similar than they appear in the 2-dimensional representation generated from CCA integration.

Lastly, we used miloR to evaluate the differences in cell type abundances within cell neighborhoods. The analysis indicated that several neighborhoods within the clusters of naïve T cells, CD8 memory, and CD8 tissue-resident memory had an increased proportion of cells coming from CIE affected dogs, while several other neighborhoods within γδ and effector CD8 T cell clusters had a reduced proportion of cells coming CIE affected dogs (**Supplementary Figure 7F**). To further investigate this potential shift in T cell subsets, we evaluated the ratio of GZMA^high^ T cells (c0, c2, c3, and c6) to IL7R^high^ T cells (c1, c4, c5, c7, c8, and c9). This analysis indicated that CIE dogs tended to exhibit an increased proportion of IL7R^high^ T cells (putative recently circulating T cells) and reduced abundance of GZMA^high^ T cells (putative tissue resident T cells) (P value 0.08, **Supplementary Figure 8D**). Further investigation using larger samples sizes is necessary to confirm the existence of a CIE associated skew in T cell subtype abundances. Overall, our analysis provides a high-resolution depiction of T cells present in the canine duodenal mucosa and demonstrates preliminary evidence of CIE associated alterations in T cell subtype proportions.

### Single-cell RNA sequencing reveals marked heterogeneity among duodenal epithelial cells and CIE associated changes to enterocyte transcriptomes

**Figure 5:**
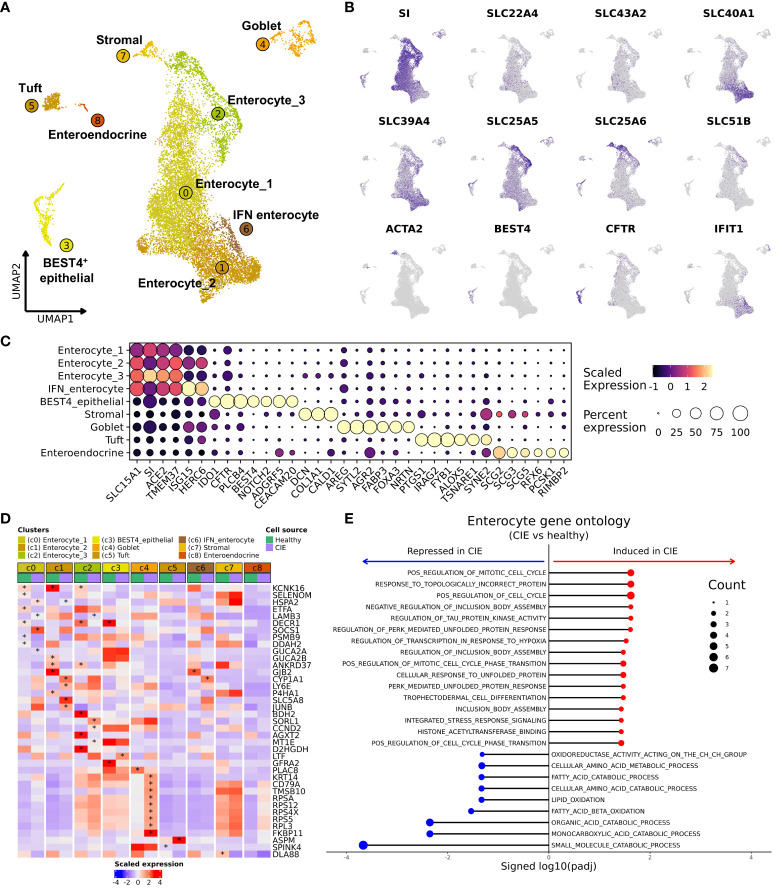
Single cell RNA-seq reveals the cellular heterogeneity within duodenal epithelial and shows markedly reduced expression of KCNK16 in CIE enterocytes. **(A)** UMAP representation of 12,457 duodenal epithelial cells. Epithelial cell subtypes are labelled based on identity, with increasing numbers corresponding to smaller relative contribution to the epithelial fraction. **(B)** Feature plots of selected genes used to define the duodenal epithelial populations. **(C)** Dot plot depicting scaled gene expression in the 9 epithelial cell subtypes. **(D)** Heatmap depicting scaled average expression within each epithelial cluster separated by condition (“CIE” or “Healthy”). Asterisks indicates statistically significant difference in expression between condition adjusted P value < 0.1 and | log2(Fold change) | > 1. The asterisk is located on the condition that had significantly higher expression. **(E)** Lollipop chart representing results of gene ontology analysis comparing enterocytes (c0, c1, c2) from CIE dogs to those from healthy dogs (red dots indicate pathways overrepresented in CIE, blue dots indicate pathways overrepresented in healthy). Count (size of dot) indicates the number of features mapping to the gene set.

Investigation of the most abundant cell type, epithelial cells, revealed the presence of 4 enterocyte populations (c0–2, c6), BEST4^+^ epithelial cells (c3), goblet cells (CLCA1^+^/AGR2^+^; c4), tuft cells (TRPM5^+^/IRAG2^+^/IL17RB^+^; c5), enteroendocrine cells (CHGB^+^/ADGRG4^+^; c8), and a cluster of stromal cells (c7) (**Figures 5A-C**; **Table 2**; **Supplementary Figure 9A**; **Supplementary Data 8**). Cell type annotations were made using gene signature enrichment scoring and reference mapping to the Human Gut Atlas (**Supplementary Figures 9B-D**) (32). Enterocytes (SI^+^) were further annotated as 4 distinct populations with smallest cluster, IFN-enterocytes, being enriched in interferon-associated gene signatures (ISG15, RSAD2, IFIT1). The remaining enterocyte clusters were divided based on the expression of solute carrier family members, where enterocyte 1 exhibited highest expression of transporters SLC22A4 and SLC43A2, enterocyte 2 had high expression of SLC40A1, which has a role in iron export, and enterocyte 3 was defined by expression of mitochondrial transporter SLC25A5 and anion transporter SLC25A6 (**Figure 5B**) (59, 60). A small population of cells enriched in expression of genes associated with tissue structure and integrity (ACTA2, THY1, DCN, COL1A1) were classified as stromal cells (61). Lastly, BEST4^+^ epithelial cells were annotated based on the expression of BEST4, MT1E, GUCA2A, and CFTR which is consistent with the cell type identified in humans and are predicted to be involved in gut regulatory processes (e.g., motility, satiety) and the absorption of dietary heavy metals (26).

Next, we completed differential abundance analysis and found a reduction in the proportion of tuft cells in dogs with CIE relative to healthy (P value = 0.057, CIE = 1.84 ± 1.70%, Healthy = 5.65 ± 2.02%; **Supplementary Figure 9E**). To further investigate CIE associated changes, we completed DGE analysis across all epithelial cells. The analysis identified CYP1A1 and HSPA2 expression to be increased, as well as the downregulation of 13 features (KCNK16 most significant) in dogs with CIE (**Supplementary Figure 9F**). To localize which cells were driving differential expression, we next repeated DGE analysis within each enterocyte cluster. In total we identified 157 DEGs across the 8 clusters evaluated (enteroendocrine cells excluded due to too few cells). Visualization of the results illustrated that one of the enriched genes, CYP1A1 (cytochrome P450 family 1 subfamily A member 1) was overexpressed in CIE within enterocyte 2 and IFN-enterocytes (c1 and c6), while the KCNK16 (potassium two pore domain channel subfamily K member 16) was downregulated within enterocyte 1, 2, and 3 (c0, c1, and c2) (**Figure 5D**). With enterocytes (c0, c1, c2) identified as having the most pronounced transcriptomic changes, we then pooled the three cell types together and complete DGE followed by GSEA. The analysis revealed an enrichment of terms associated with responses to misfolded proteins, stress responses, and cell cycle regulation in cells from CIE dogs. Repression of terms associated with catabolic processes and cellular metabolism was also appreciated (**Figure 5E**), suggesting the enterocyte clusters may be undergoing stress responses and altering their metabolic properties. Overall, our analysis identified marked diversity within canine duodenal epithelial cells and highlighted heightened stress responses and altered metabolism in CIE enterocytes.

## Discussion

Chronic inflammatory enteropathy (CIE) is the most common reason for chronic diarrhea in dogs (2, 3), yet its pathogenesis is poorly understood, preventing specific therapeutic interventions and evidence-based clinical management. The present study applied single-cell RNA sequencing (scRNA-seq) to interrogate cell populations present in duodenal mucosal biopsies from healthy dogs and dogs with CIE. This approach enabled elucidation of the cellular diversity and transcriptomic programs present amongst cells of the canine duodenal mucosa. Analysis revealed disease associated transcriptomic changes within epithelial cells and myeloid cells, while also highlighting a potential shift in the relative proportions of T cell subtypes. Our analysis resulted in the generation of a reference dataset of canine duodenal cell type gene signatures that can be applied to further study the cellular and molecular mechanisms at play in chronic intestinal conditions affecting dogs.

Comparison of myeloid cells within the duodenal mucosa between the two groups of dogs revealed significant transcriptomic differences in duodenal neutrophils of CIE affected dogs. We found that cells isolated from CIE samples were the source of IL1A and SOD2 overexpression, as identified through DGE analysis. This is consistent with findings of prior studies which have identified higher S100A12 concentrations in duodenal biopsies via ELISA (62) and in fecal samples from dogs with CIE compared to healthy controls (63). S100A12 has been identified to be specific for canine neutrophils (19). Other studies have identified the degree of neutrophilic inflammation in the lamina propria as a useful histopathologic parameter for CIE evaluation, showing a moderate correlation with CIE disease severity (CCECAI) (11). Although our analysis did not identify a statistically significant increase in neutrophil relative abundances, most of the differentially expressed genes were associated with neutrophils. Given the limited number of myeloid cells in the dataset, and the outsized contribution to the neutrophil signature by a single CIE dog, further investigation is required. A strong neutrophilic duodenal infiltrate may signify a distinct disease entity; larger studies will be needed to prove or disprove that theory and determine the stimuli for neutrophil recruitment and activation. Our results nevertheless underscore the benefit of gene expression profiling in tandem with comparison of cell proportions.

In line with results from IHC and flow cytometric evaluation of canine duodenal biopsies (13, 40, 64, 65) we found CD8^+^ T cells were the predominant T cell subtype. Nevertheless, canonical cell surface markers (CD4, CD8) were generally expressed at low levels and did not drive clustering when we applied Seurat’s CCA integration approach. However, through the use of an alternative integration approach, single-cell Variational Inference (scVI), we were able to generate a low-dimensional representation of the data that highlighted the division of T cells based on CD4/CD8 expression. In the CCA integrated T cell dataset, we found that T cells were better described based on expression of genes indicative of tissue residency within the intestinal mucosa (GZMA^high^ T cells) or a more recently emigrated state (IL7R^high^ T cells). Based on gene expression profiles, we propose that the cluster of IL7R^high^ T cells identified in our dataset correspond to T cells present within mucosal microvasculature at the time of biopsy, as well as T cells recently arriving in the duodenal mucosa from circulation. The cells within the GZMA^high^ cluster broadly expressed features associated with effector functions and integrins required for maintenance within mucosal tissue, suggesting the cluster represents tissue resident T cells. While subclusters within IL7R^high^ T cells were readily identified, we found substantial overlap between the GZMA^high^ T cell subpopulations. This phenomenon of broad transcriptomic similarity among T cell subtypes has been previously described murine intestinal and airway T cells (66, 67). Our results shed light on the complex and heterogeneous intestinal T cell landscape beyond what has been identified using flow cytometric and IHC based analyses (14, 40).

Additionally, our analysis of canine duodenal mucosal lymphocytes provided evidence that IL7R^high^ T cells were overrepresented with a concurrent underrepresentation of GZMA^high^ T cells in the duodenal mucosa of CIE dogs relative to healthy dogs. Single-cell RNA-seq investigation of Celiac disease has similarly identified depletion of a subset of natural (unconventional) intraepithelial CD8^+^ T cells in duodenal biopsies from affected individuals, with concurrent expansion of cytotoxic CD8^+^ T cell (21). It is possible that we observed a similar phenomenon in our dataset and that the shift in proportions of IL7R^high^ and GZMA^high^ T cells is relevant to understanding CIE pathogenesis. Unsurprisingly, previous groups have found no change in the density of CD3^+^ cells in duodenal biopsies from dogs with CIE before and during remission (41, 68), which is likely due to the non-specific nature of CD3. Future studies applying the cell subtype gene signatures of the IL7R^high^ and GZMA^high^ T cell populations reported here are warranted to further investigate their role in CIE pathobiology. Quantifying proportions of these cell subtypes before and after intervention could provide further support to the relevance of these cell populations in CIE dogs.

Through use of scRNA-seq, we were able to compare the transcriptomes of intestinal epithelial cells from healthy dogs and dogs with CIE. This approach highlighted differential expression of several genes, with the most significant change being a reduction (in CIE relative to healthy) in the expression of KCNK16, a gene encoding a 2-pore potassium channel, within epithelial cells. Potassium channels play essential roles in epithelial cell homeostasis in other species, regulating membrane voltage, cell volume, and cell proliferation (69). Mice lacking the gene KCNK9 were found to be particularly susceptible to DSS-induced colitis (70). Our results indicate that a lack of duodenal KCNK16 expression may play a role in CIE pathogenesis, but it is also possible that the differential expression was due to breed differences, as our control population were all beagles and the CIE dogs consisted of 4 non-beagle purebred dogs. One of the top upregulated genes, CYP1A1, is an effector molecule of the aryl hydrocarbon receptor in mice and humans (71, 72). Excessive CYP1A1 signaling has been associated with intraepithelial lymphocyte death (72) and dysregulation of enterocyte proliferation and differentiation (73) in mice, making this an interesting gene for further study in dogs with CIE. Pathway analysis of enterocytes pointed to altered metabolic activities and responses to protein inclusions bodies. These findings highlight potential important contributions of intestinal epithelial cells to CIE pathogenesis.

Although the data reported here serves as a starting point for further investigation into the complex duodenal mucosal environment, the data and analysis performed here are not without limitations. The two patient populations were not matched in terms of breed, neuter status, or size. Male rat intestine showed a greater inflammatory reaction to stress than female (74), but the influence of testosterone has not been explored in canine intestinal cells. These discrepancies, as well as differences in the environment of the colony-house beagles compared to client-owned dogs in home environments may have contributed to the differences between healthy and CIE dogs in this study. Future studies should include a balanced mix of sexes, breeds, and sizes, housed in similar fashion, in each population. Our endoscopic biopsies preferentially sampled superficial duodenal tissues (i.e., villus tips), which could have led to bias toward the intraepithelial cell populations, with lesser contributions from the lamina propria. This may explain our inability to resolve Paneth cells, which are typically very rare cells in scRNA-seq datasets (26, 75). The tissue dissociation protocol and Ficoll density gradient centrifugation may have preferentially enriched for or depleted certain cell types (76, 77), thus the proportions documented here may not exactly represent the proportions present in duodenal tissue *in vivo*. Furthermore, gene expression profiles of cells may have been influenced by the dissociation process, which has been reported to enrich for stress-response-related genes (77). In our dataset, this was most evident within the enterocytes, which had higher-than-expected percentage of mitochondrial reads. Lastly, we documented wide variability in cell type proportions isolated from individual CIE dogs with less variability among the 3 healthy beagle dogs. The heterogeneity observed within the CIE samples likely reflects the diverse pathogenesis of this condition in dogs, which is more accurately a syndrome rather than an isolated disease entity but may have also been influenced by the breed differences within that group.

The findings from this study provide important new insights into the pathogenesis of canine CIE, implicating enterocytes, neutrophils, and T cell subtypes in the disease process. In addition, this work provides an important resource for the study of canine immune and intestinal cell types, as the dataset includes a comprehensive catalog of cells in the duodenum of dogs, which can be used to support future investigations.

## Data availability statement

Raw sequencing data and cell by gene countmatrices are available on the NCBI Gene Expression Omnibus database (GSE254005). The annotated dataset is available for browsing on the UCSC Cell Browser (https://cells.ucsc.edu/?ds=canine-duodenum-cie) (35). Processed data (Seurat RDS objects), analysis code, and software versions are available on Zenodo and at https://github.com/dyammons/canine_duodenal_atlas (24). Any additional data requests can be made by contacting the corresponding author.

## Ethics statement

The animal studies were approved by Colorado State University Clinical Review Board. The studies were conducted in accordance with the local legislation and institutional requirements. Written informed consent was obtained from the owners for the participation of their animals in this study.

## Author contributions

AM: Writing – original draft, Writing – review & editing, Conceptualization, Data curation, Funding acquisition, Investigation, Methodology, Project administration. DA: Data curation, Formal analysis, Investigation, Methodology, Resources, Software, Supervision, Visualization, Writing – original draft, Writing – review & editing. SD: Conceptualization, Formal analysis, Funding acquisition, Investigation, Project administration, Resources, Supervision, Writing – review & editing. ML: Funding acquisition, Project administration, Resources, Supervision, Writing – review & editing.
