# Supplementary material for: Single cell transcriptomic analysis of the canine duodenum in chronic inflammatory enteropathy and health
Source: Front Immunol. 2024 Jun 12;15:1397590. doi: 10.3389/fimmu.2024.1397590 (PMC11199541; doi:10.3389/fimmu.2024.1397590)
Supplement: Supplementary file 1 [file DataSheet_1.pdf]

Supplemental figures for…

Single cell transcriptomic analysis of the canine duodenum in chronic inflammatory enteropathy and health

Running title: Canine scRNA-seq duodenum

Alison C. Manchester[1]*†, Dylan T. Ammons[2]†, Michael R. Lappin[1] and Steven Dow[1,2]*

†These authors contributed equally to this work and share first authorship.

[1]Colorado State University, Department of Clinical Sciences, College of Veterinary Medicine and Biomedical Sciences, Fort Collins, CO, USA

[2]Colorado State University, Department of Microbiology, Immunology and Pathology, College of Veterinary Medicine and Biomedical Sciences, Fort Collins, CO, USA

***Correspondence:**
Alison C. Manchester; Alison.manchester@colostate.edu

Steven Dow; steven.dow@colostate.edu

Supplemental tables:

Supplemental table 1: Gene symbols and literature citations to support cell type classifications

Supplemental table 2: Antibody panel used for flow cytometric analysis.

Supplemental table 3: Conclusions of histopathologic analysis of endoscopically obtained duodenal biopsies from study dogs.

Supplemental table 4: Summary metrics from Cell Ranger alignment.

Supplemental table 5: Cell viability based on flow cytometric analysis.

Supplemental data:

Supplemental data 1: Full cell type gene signatures identified for the 7 major cell populations using the Seurat function FindAllMarkers().

Supplemental data 2: Full cell type gene signatures identified for the 31 individual cell types using the Seurat function FindAllMarkers().

Supplemental data 3: Full gene lists comparing gene expression between CIE and healthy in all cells and within each major cell type.

Supplemental data 4: Full cell type gene signatures for myeloid cell types using the Seurat function FindAllMarkers().

Supplemental data 5: Full cell type gene signatures for low-resolution T cell types using the Seurat function FindAllMarkers().

Supplemental data 6: Full gene lists comparing IL7R^{high} T cells to GZMA^{high} T cells.

Supplemental data 7: Full cell type gene signatures for high-resolution T cell types using the Seurat function FindAllMarkers().

Supplemental data 8: Full cell type gene signatures for epithelial cell types using the Seurat function FindAllMarkers().

Supplemental data column values:

Supplemental data 1, 2, 4, 5, 7, and 8:

cluster = cell type
gene = gene symbol
p_val = unadjusted P value
avg_log2FC = log2 fold change
pct.1 = percentage of cells expressing the gene in the cell type
pct.2 = percentage of cells expressing the gene in all other cells within the dataset
p_val_adj = adjusted P value
UniProt.description = description of gene from UniProt
Surfaceome.Label = indicates if gene is predicted to be on the surface
Surfaceome.Label.Source = method used to determine if gene is on surface

Supplemental data 3:

gene = gene symbol
baseMean = average of the normalized count values
log2FoldChange = log2 fold change (CIE vs healthy)
lfcSE = standard error of the log2FoldChange estimate
stat = Wald statistic
pvalue = Wald test P value
padj = Benjamini-Hochberg (FDR) adjusted P value
cellType = Cell type in which CIE was contrasted to healthy
contrast = direction of comparison [term1]_vs_[term2]; avg_log2FC is based on the contrast where log2FoldChange > 0 is up in term1 and log2FoldChange < 0 is up in term2

Supplemental data 6:

Same as Supplemental data 3, except no cellType column.

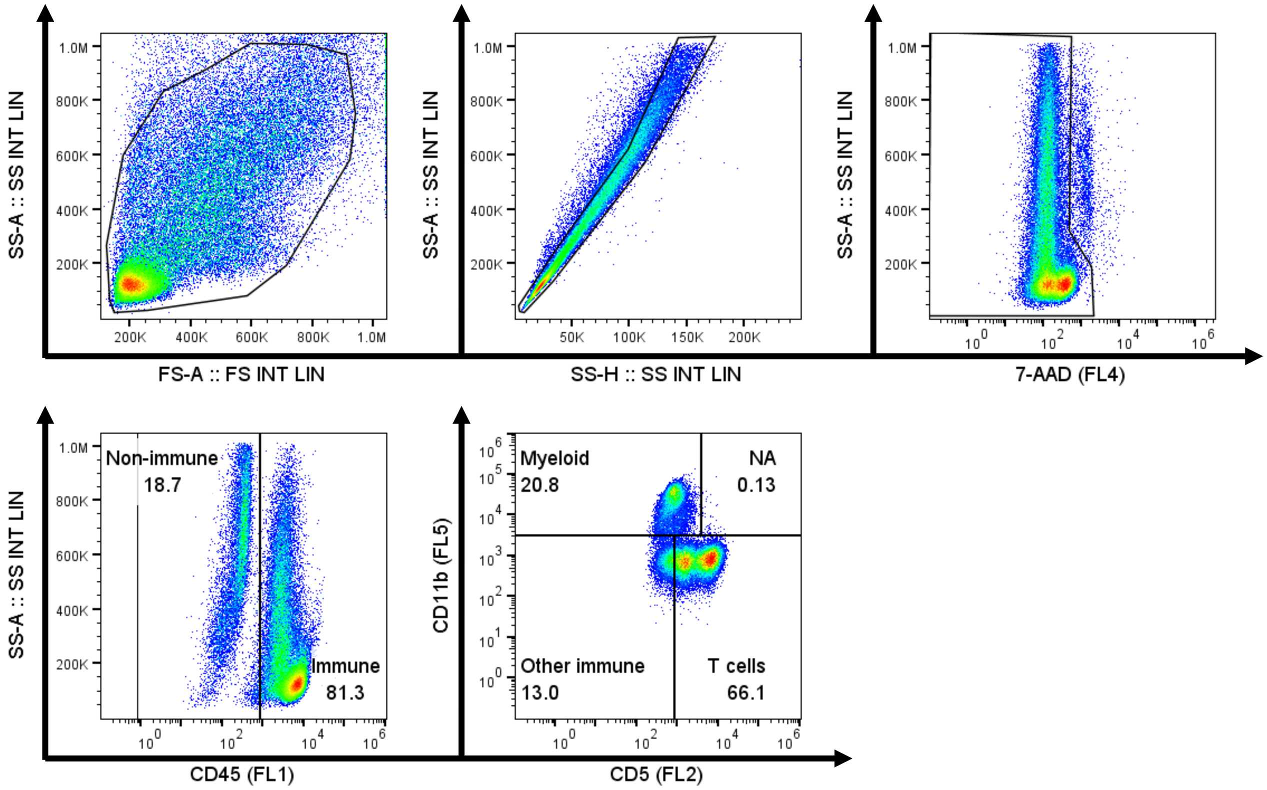

Supplemental figure 1. Flow cytometry gating scheme. Representative flow cytometric gating strategy used to analyze dissociated cells from endoscopically obtained duodenal biopsies. Representative plots depicted the data for healthy beagle “H_1”.

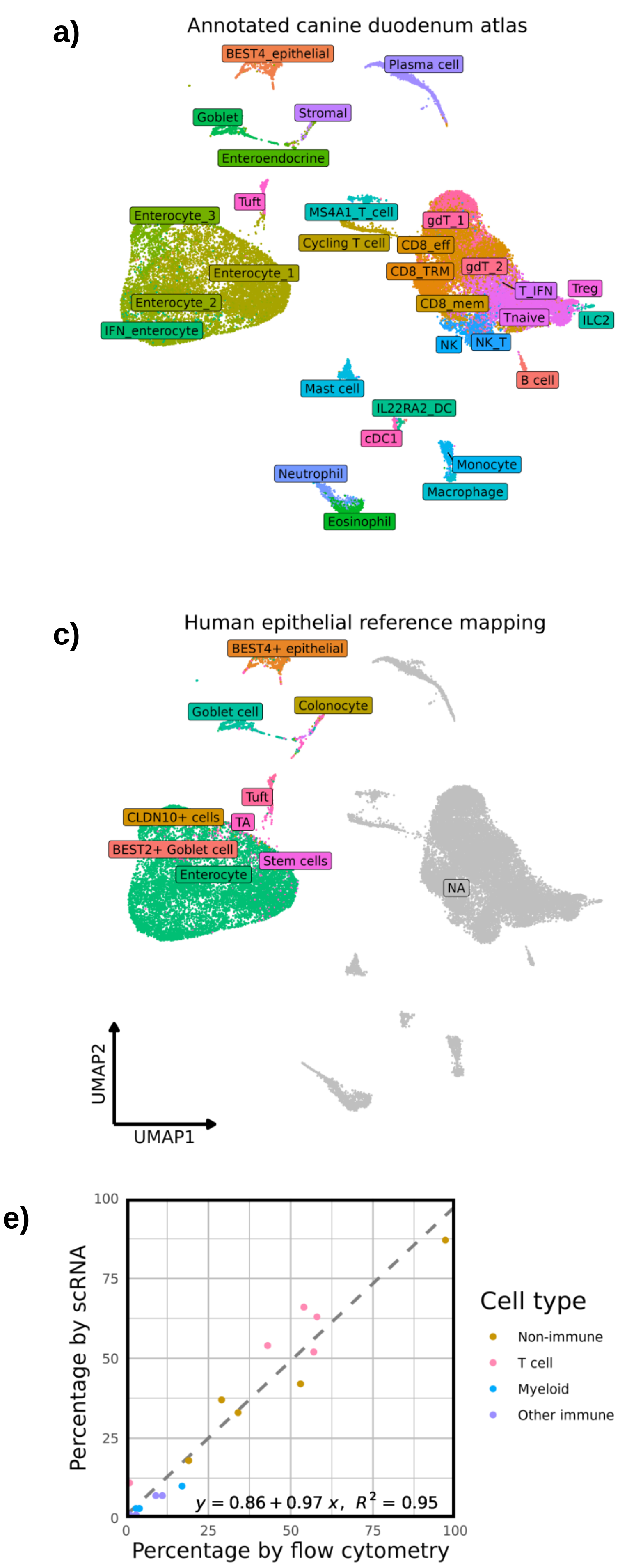

Supplemental figure 2. Supporting data for cell type annotations and flow cytometric validation. associated with primary Figure 2. UMAP representation of duodenal cells from 3 healthy dogs and 4 dogs with CIE colorized by (**a**) final cell type annotation, cell type as determined by reference mapping to (**b**) canine PBMCs, (**c**) human gut atlas epithelial subset, or (**d**) human gut atlas T cell subset. Cells annotated as “NA” were excluded from the label transfer protocol due to not being represented in the reference dataset. (**e**) Scatter plot depicting cell type percentage as determined by flow cytometry (x-axis) or single-cell RNA sequencing (y-axis).

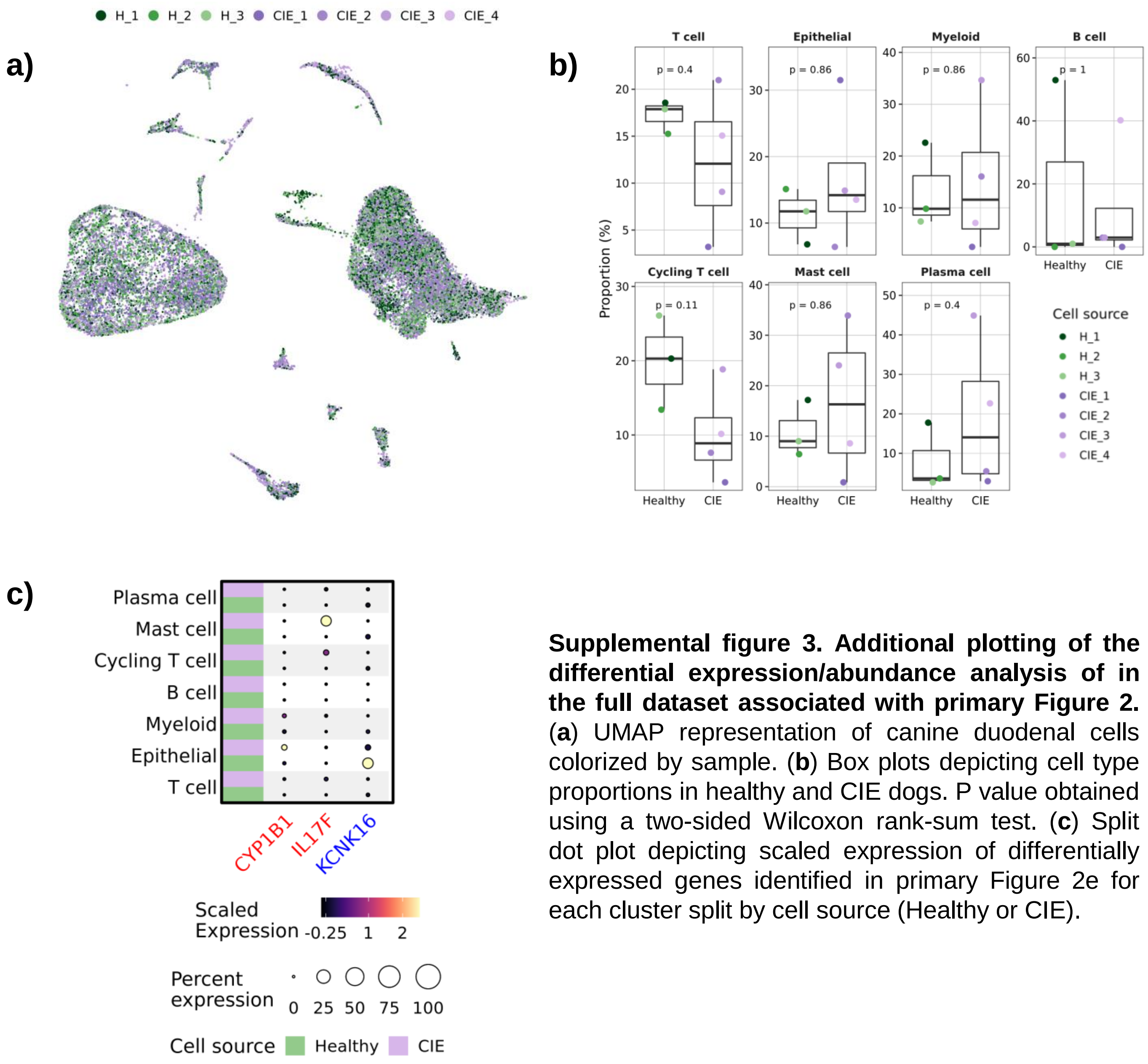

Supplemental figure 3. Additional plotting of the differential expression/abundance analysis of in the full dataset associated with primary Figure 2. (**a**) UMAP representation of canine duodenal cells colorized by sample. (**b**) Box plots depicting cell type proportions in healthy and CIE dogs. P value obtained using a two-sided Wilcoxon rank-sum test. (**c**) Split dot plot depicting scaled expression of differentially expressed genes identified in primary Figure 2e for each cluster split by cell source (Healthy or CIE).

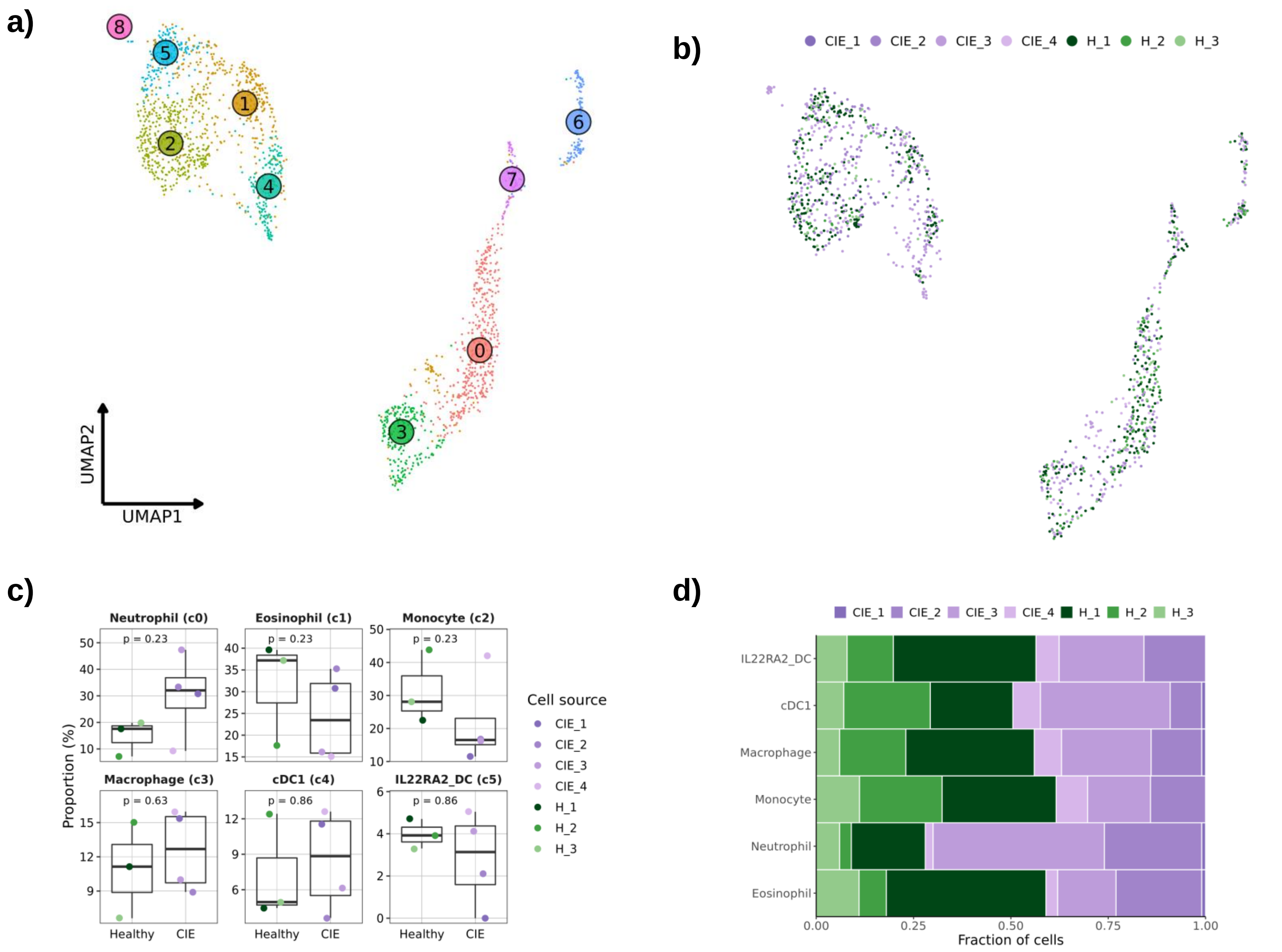

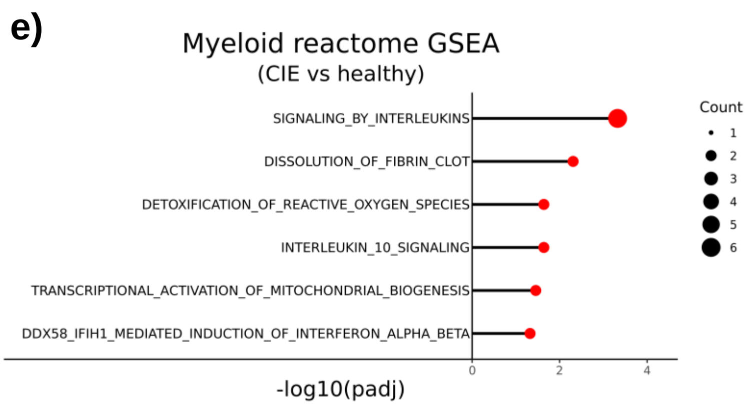

Supplemental figure 4. Additional plotting of the myeloid subset associated with primary Figure 3. UMAP representation of myeloid cells (n=1,945) colorized by (**a**) unsupervised clustering results or (**b**) by sample. (**c**) Box plots depicting cell type proportions in healthy and CIE dogs. P value obtained using a two-sided Wilcoxon rank-sum test. (**d**) Stacked bar graph depicting proportion of cells from each sample within each cell subtype. (**e**) Lollipop chart depicting the gene set enrichment results of the differentially expressed genes identified in primary Figure 3c using the Reactome database. Count (size of dot) indicates the number of features mapping to the gene set.

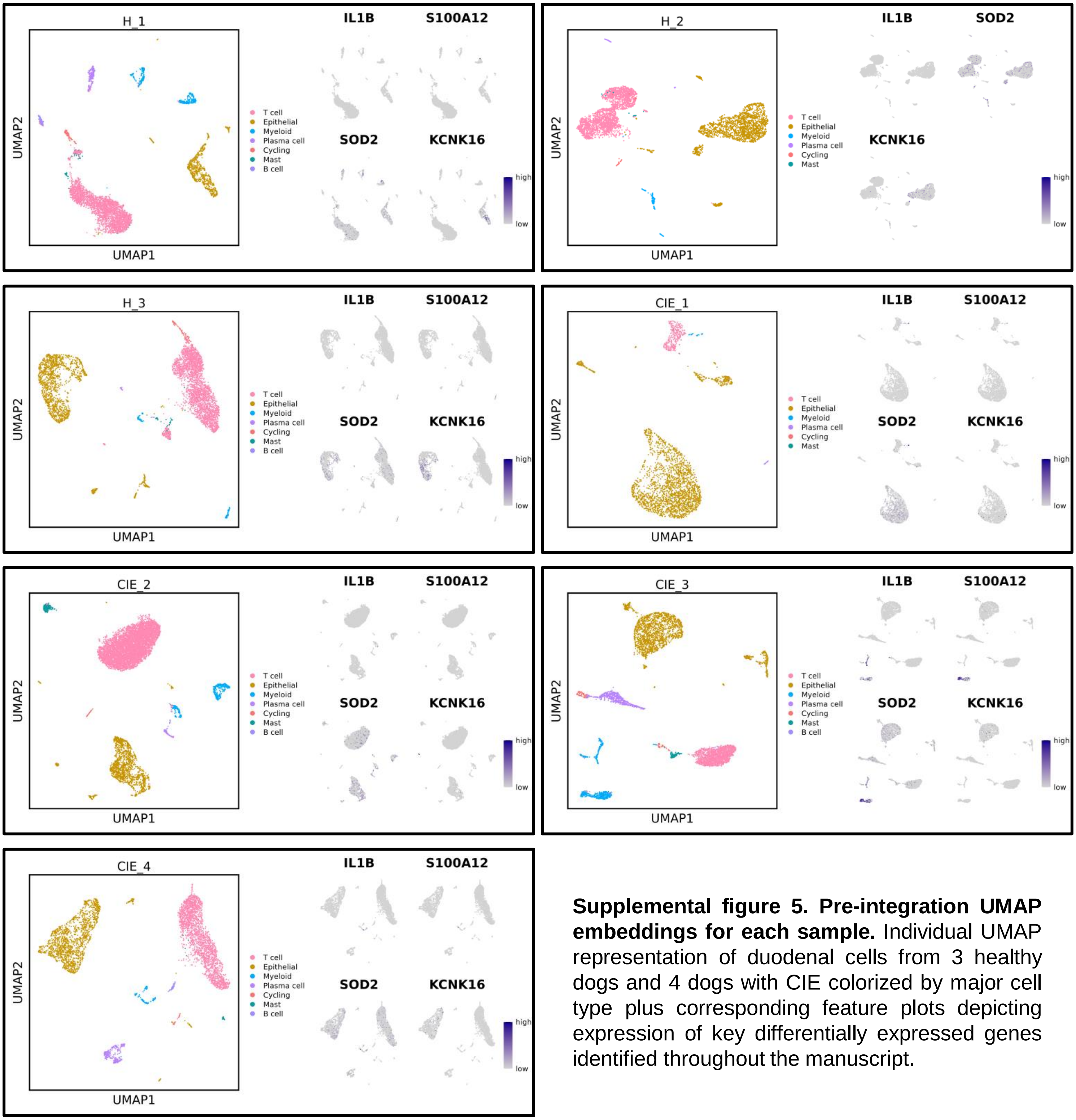

Supplemental figure 5. Pre-integration UMAP embeddings for each sample. Individual UMAP representation of duodenal cells from 3 healthy dogs and 4 dogs with CIE colorized by major cell type plus corresponding feature plots depicting expression of key differentially expressed genes identified throughout the manuscript.

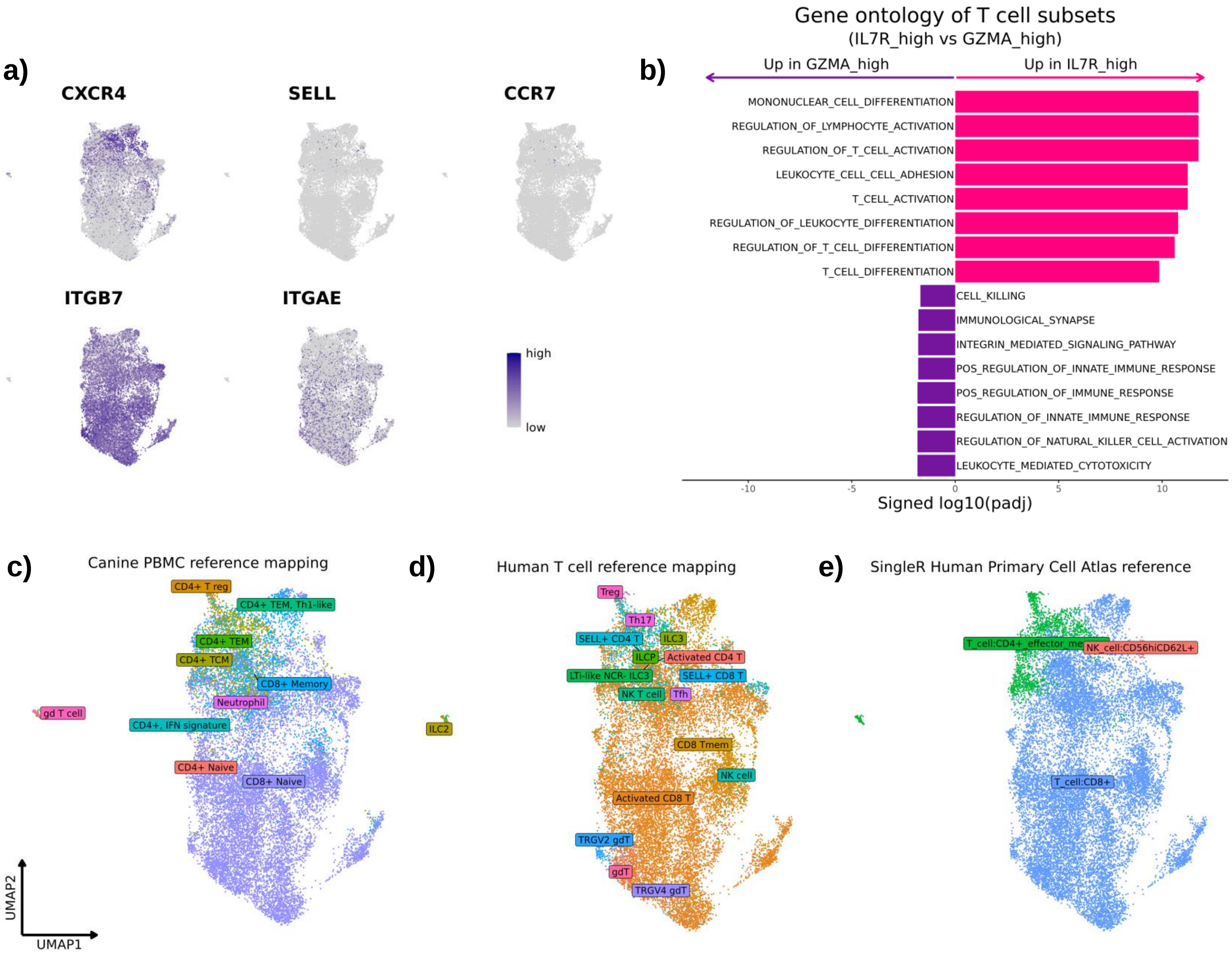

Supplemental figure 6. Additional plotting of the T cell subset associated with primary Figure 4. (**a**) Feature plots depicting expression of select genes. (**b**) Bar chart representing results of gene set enrichment analysis (GSEA) comparing IL7R^{high} to GZMA^{high} T cells (pink bars indicate pathways enriched in IL7R^{high}, purple bars indicate pathways enriched in GZMA^{high}). UMAP representation colorized by cell type as determined by reference mapping to (**c**) canine PBMCs, (**d**) human gut atlas T cell subset, and (**e**) SingleR annotation using Human Primary Cell Atlas.

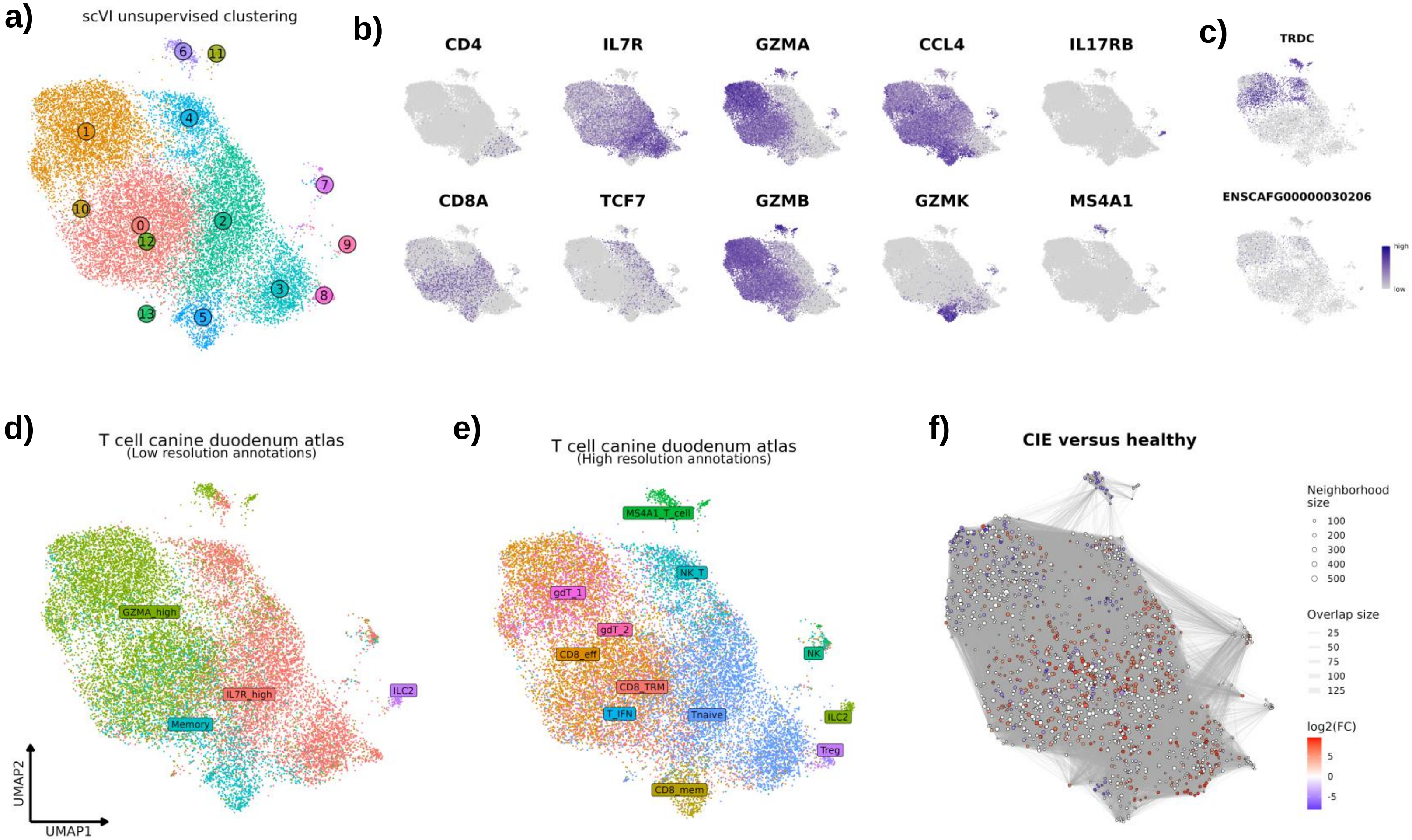

Supplemental figure 7. Results of single cell Variational Inference (scVI) integration approach on canine duodenal T cells from 3 healthy beagles and 4 client-owned dogs with CIE. (**a**) UMAP representation colorized by unsupervised clustering of scVI integrated data. (**b**) Feature plots depicting expression of select genes associated with the clustering of the T cell subtypes based on scVI analysis. (**c**) Feature plots depicting expression of TRDC and ENSCAFG00000030206 (TRGC2 ortholog) in only the healthy samples. To obtain TRDC expression the three healthy samples were aligned to an additional genome, ROS_Cfam_1.0, in which TRDC is annotated. UMAP representations colorized by (**d**) transferred cell type annotation described in primary figure 4a and (**e**) transferred cell type annotation described in primary Figure 4d. (**f**) UMAP representation of cell neighborhoods obtained through miloR. Neighborhoods that are significantly differentially abundant (spatialFDR P value < 0.2) between conditions (CIE versus healthy) are colored red (Overrepresented in CIE) or blue (Underrepresented in CIE). Any neighborhood that is colored white did not reach the spatialFDR P value threshold and is considered nonsignificant.

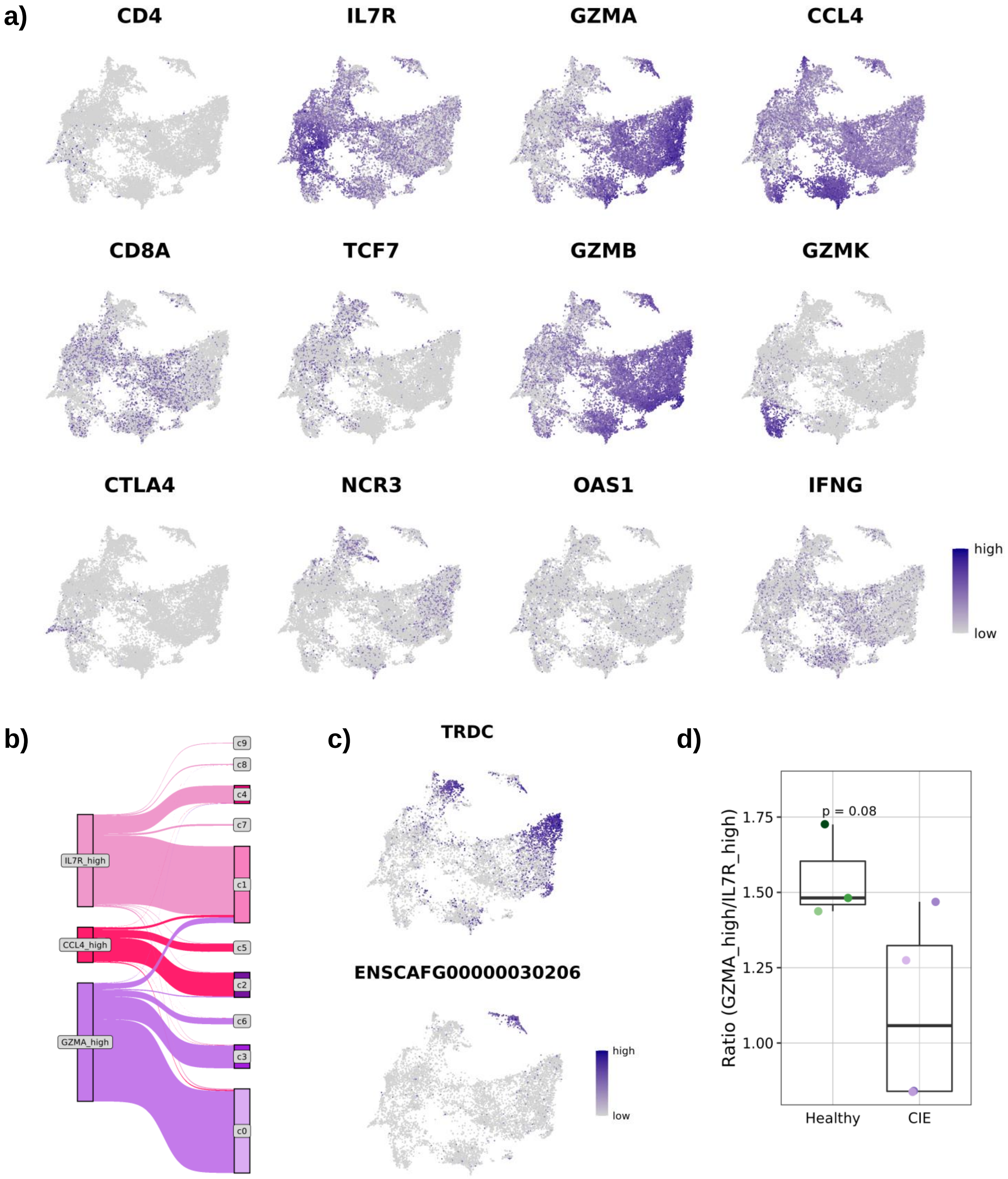

Supplemental figure 8. Additional plotting of the T cell subset associated with primary Figure 4. (**a**) Feature plots depicting expression of select genes. (**b**) Sankey plot illustrating the relationship between IL7R^{high} T cells (putatively annotated as non-resident), GZMA^{high} T cells (putatively annotated as tissue-resident), CCL4^{high} T cells and the high-resolution clusters. (**c**) Feature plots depicting normalized expression of TRDC and ENSCAFG00000030206 (TRGC2 ortholog) in only the healthy samples. To obtain TRDC expression the three healthy samples were aligned to an additional genome, ROS_Cfam_1.0, in which TRDC is annotated. (**d**) Box plot depicting the proportion of GZMA^{high} T cells (putatively annotated as tissue-resident) to IL7R^{high} T cells (putatively annotated as non-resident) in healthy and CIE dogs. P value obtained using a two-sided Wilcoxon rank-sum test.

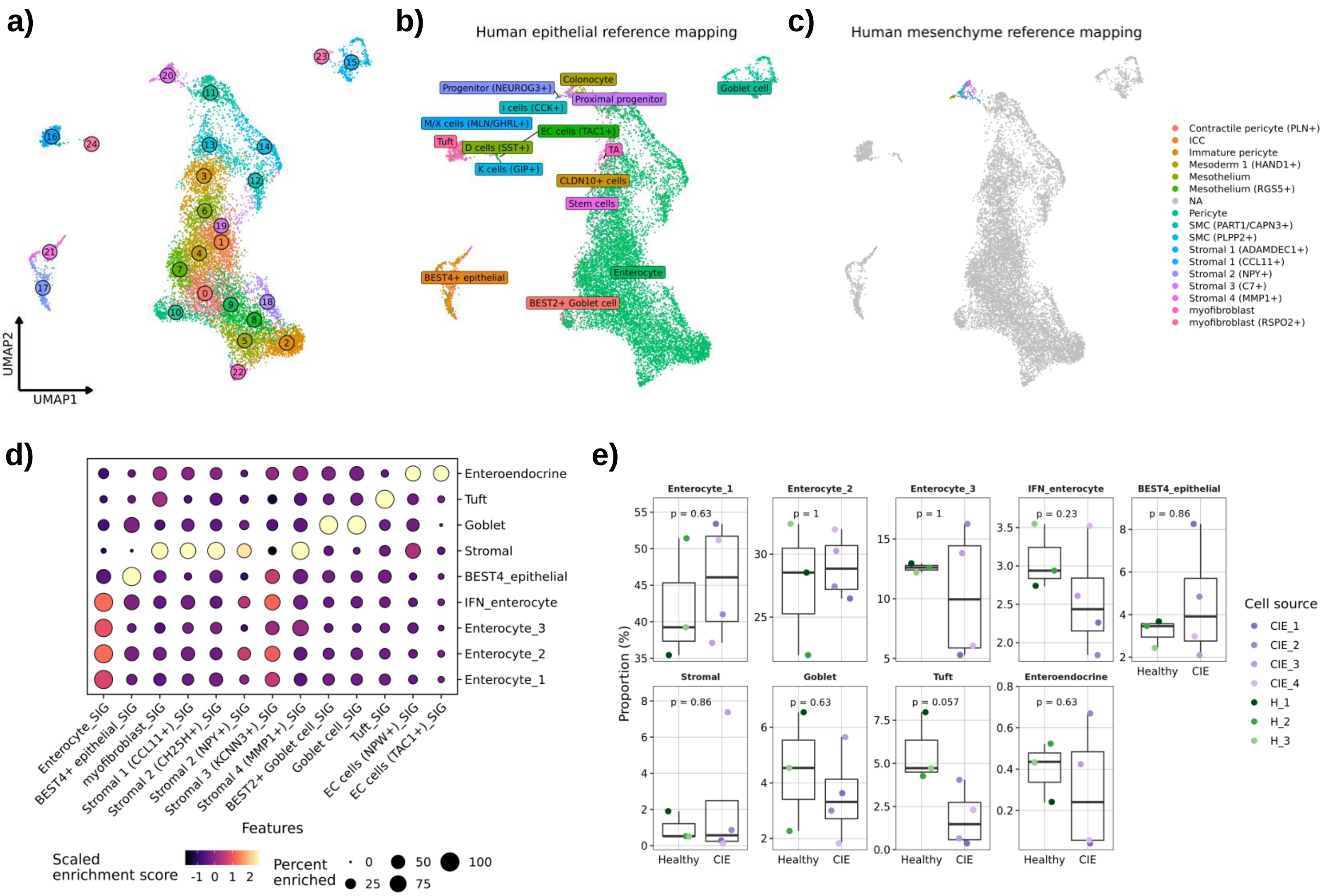

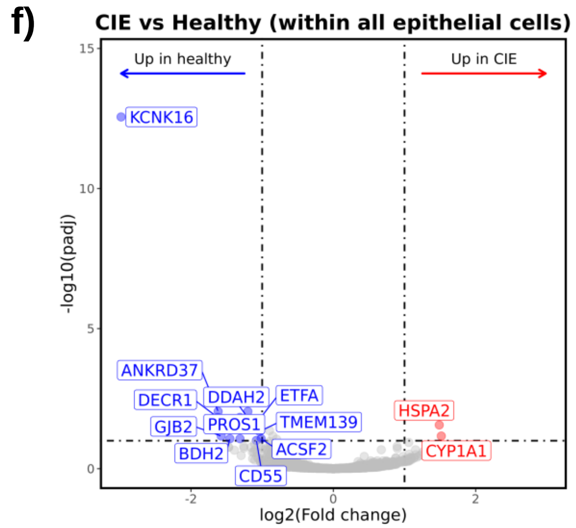

Supplemental figure 9. Additional plotting of the epithelial subset associated with primary Figure 5. UMAP representation colorized by (**a**) unsupervised clustering results or cell type annotation as determined by reference mapping to (**b**) human gut atlas epithelial subset or (**c**) human gut atlas mesenchyme subset. Cells annotated as “NA” in the mesenchyme annotated dataset were excluded from the label transfer protocol due to not being represented in the reference dataset. (**d**) Dot plot depicting gene signature enrichment using signatures from the human gut atlas. (**e**) Box plots depicting epithelial cell type proportions in healthy and CIE dogs. P value obtained using a two-sided Wilcoxon rank-sum test. (**f**) Volcano plot depicting results of pseudobulk differential gene expression analysis across all epithelial cells using DESeq2.
